# Effect of mesh sizes on the mechanical properties of stainless-steel wire mesh/glass fiber reinforced hybrid composite laminates for aerospace applications

**DOI:** 10.1038/s41598-025-20038-1

**Published:** 2025-10-15

**Authors:** Juveriya Sayyed, Aashish Ramprasad, Yogeesha Pai

**Affiliations:** Department of Aeronautical and Automobile Engineering, Manipal Institute of Technology, Manipal Academy of Higher Education (MAHE), Manipal, 576 104 Karnataka India

**Keywords:** Stainless steel wire mesh (SSWM), Glass fiber reinforced polymer (GFRP), Hybrid composite laminates, Fiber metal laminates (FMLs), Mechanical characterization, Aerospace composites, Engineering, Materials science

## Abstract

This study investigates the effect of stainless-steel wire mesh (SSWM) size on the mechanical performance of glass fiber–SSWM hybrid composites for aerospace application. Laminates were fabricated by embedding AISI 304 steel meshes (10 to 120 openings per inch) and bi-directional glass fibers in epoxy using hand layup and compression molding. Mechanical tests showed mesh size strongly affects properties: Mesh 120 achieved the highest tensile strength (539.19 MPa) with low void content (0.49%), suitable for tensile-critical parts; Mesh 20 had the highest flexural strength (487.97 MPa) due to its heavier mesh weight and low voids (0.53%), ideal for load-bearing panels; Mesh 40 offered the best balance of impact resistance (1.715 J), tensile (409.10 MPa), and flexural (339.78 MPa) strengths, supporting multifunctional structures. Finer meshes (80, 120) exhibited improved ductility and damping for vibration-sensitive applications. SEM analysis revealed strong interfacial bonding and crack-bridging in Mesh 40, while Mesh 120 showed microcracking and fiber pull-out, indicating trade-offs between tensile and impact performance. Void content ranged from 0.49% to 1.93%, confirming high manufacturing quality. These findings emphasize the critical role of mesh geometry in optimizing hybrid composite performance and suggest further work on advanced fabrication methods and durability testing.

## Introduction

The aerospace industry’s usage of composite materials has grown from secondary structures to main airframe components over the past decades. Following initial applications in control surfaces and rear sections (Airbus A320 in 1988, Boeing 777 in 1995), composite materials are today employed in most of the new airframe structures, as seen in the all-composite Boeing 787 (2011) and Airbus A350. This shift has been induced by an increased strength-to-weight ratio and corrosion resistance compared to traditional aluminum airframes^1^. Of these materials, Glass Fiber Reinforced Polymer (GFRP) has especially emerged as an essential aerospace component, offering 20–30% weight savings without compromising structural integrity in applications from fuselages and landing gears to interior structures and tail spoilers^2^. On further development of this material, Fiber Metal Laminates (FMLs) provide the advantages of metallic and composite systems through layered structures such as GLARE (glass/aluminum), ARALL (aramid/aluminum), and CARALL (carbon/aluminum). These hybrid materials exhibit enhanced fatigue life and damage tolerance over monolithic composites, and they are well-renowned for fatigue-critical airframe structures. Their tensile, flexural, and impact mechanical properties are of active interest now as a key focus area for the aerospace sector in an effort to maximize next-generation lightweight structures^3,4^. Hybrid material systems have evolved recently, and metal wire meshes have been integrated into glass fiber reinforcement to enhance mechanical performance. The strategic integration of wire mesh structures within glass and carbon fiber laminates significantly improves the tensile strength and flexural stiffness without compromising the energy-absorbing properties of the metal^5^. Recent studies have extensively evaluated hybrid composites for improved structural performance. Jamil et. al^6^. investigated steel-reinforced laminates and reported notable structural improvements. Their results showed that the incorporation of steel fibers and wire mesh enhanced flexural strength by 9–10% and strain energy by 23–31% over unreinforced laminates under three-point bending tests. The reinforcements were found to inhibit crack propagation at the steel layer, highlighting their potential for lightweight armored vehicles with improved ballistic resistance. Based on these findings, Choudary et. al^7^. performed a systematic comparison of reinforcement materials and concluded that glass fiber–reinforced composites combined with stainless steel mesh exhibit superior performance. Specifically, flexural strength exceeded that of glass, carbon, and jute fibers, while tensile strength and impact resistance were improved by 15–20% and 25–30%, respectively, compared with conventional glass fiber–reinforced plastics. Said et al.^8^ demonstrated that double and triple hybridization with metallic mesh can increase un-notched and notched strength by up to 30%, thereby addressing structural integrity requirements. In a subsequent study, Said et al.^9^ highlighted that strategic steel mesh hybridization improved damage tolerance by 25% and contributed to vibration damping through energy dissipation—an attribute critical for cabin panel applications. Sadoun et al.^10^ reported a 20–35% increase in tensile strength alongside a ~ 15% weight reduction using optimized mesh architectures, directly supporting fuel-efficiency objectives. Additionally, Said et al.^11^ noted a 40% improvement in shear strength, contributing to enhanced fracture toughness for high-performance structures. Integrating stainless steel wire mesh (SSWM) with glass fibers therefore addresses multiple design requirements by delivering superior structural integrity, vibration resistance, and lightweight performance. The key research objectives include quantifying these mechanical enhancements and tailoring them for application-specific designs. Such improvements render hybrid composites highly suitable for aerospace and automotive applications where an optimal balance of strength, stiffness, and damage resistance is essential. Notably, comparative evaluations also revealed that SSWM composites outperform aluminum-mesh counterparts in terms of toughness and energy absorption capacity^12^.

Following stainless steel’s proven capability, new research has investigated various metal mesh composites with specific properties. Szczepanik et al.^13^ research involving hot closed-die forging of steel mesh-reinforced aluminum matrix composites proved the possibility of making light-weight components with high bending strength (431–478 MPa) without the formation of brittle Fe-Al phase. Concurrently, Fritzen et. al^14^ introduced A periodic Voronoi tessellation technique to produce 3D microstructures of particle-reinforced composites (Al-SiC) to allow efficient finite element analysis of thermo-elastic properties. Their technique provided precise predictions even with coarse discretizations (≈ 10,000 nodes) and showed that effective ISOtropic properties stay within Hashin-Shtrikman bounds for up to 80% volume fractions. Krishnasamy et al.^15^ showed that Kevlar/brass wire mesh composites with 3.0 vol% cubic boron nitride particles in B/K/B/K stacking sequence exhibited maximum mechanical performance, exhibiting 35% enhancement in impact resistance and load capacity compared to other layups. As confirmed by ASTM-standard testing, the improved delamination toughness and energy absorption of the hybrid system render it ideal for armor and aerospace use where combined toughness and lightweight functionalities are needed.

Apart from mechanical properties, Tugirumubano et al.^16^ work on hybrid stainless steel-copper wire mesh/CFRP laminates achieved an excellent electromagnetic interference shielding effectiveness of 131.6 dB, for which absorption losses accounted for 82% of the overall shielding performance. By incorporating prepreg carbon wefts into alternating metal wire warp meshes, the new plain-weave structure maintained mechanical properties and enhanced EMI protection over traditional materials. This is consistent with Truong et al.^17^ research that stainless steel wire mesh reinforcement of carbon fiber composites provides better tensile performance, as it improves initial stiffness, peak load, and ultimate strain by 20–30% over non-hybrid counterparts. The research established A reliable model of load-strain behavior prediction that was tested systematically in 18 configurations with different fiber orientations (0°/90°) and layer stacking sequences (2–4 CF layers, 1–3 SM layers). The research illustrates how metal mesh hybridization, especially with stainless steel, has progressed from optimizing individual material properties to allowing truly multifunctional composite systems for use in advanced aerospace, automotive, and protective applications.

More recently, the dynamic and aging properties of hybrid composites have been investigated in greater depth, which are very important in applications for aerospace under fluctuating and cyclic loads. Agarwal et al.^18^ examined the mechanical and damping properties of thin quasi-isotropic hybrid carbon-Kevlar/epoxy intraply composites and found that tensile strength decreases by up to 15% due to aging but increases by 20–25% in damping properties as a result of enhanced interfacial friction, emphasizing the need for long-term performance under vibrational stress. Also, Shaikh et al.^19^ studied the effect of moisture on quasi-isotropic Kevlar-basalt/epoxy hybrid composite, observing 10–12% reduction in flexural strength and an increase in damping ratio by 30% following moisture absorption, depicting the importance of taking environmental influence on vibration response into account. Namrata et al.^20^ examined the effects of aging on quasi-isotropic basalt fiber-reinforced polymer composites and found a 5–8% loss in natural frequency and an enhancement in damping ratio by 15% after 500 h of aging, which is crucial for determining vibration-induced fatigue in aerospace structures. In addition, Pai et al.^21^ investigated the influence of aramid fabric orientation angle on basalt-aramid/epoxy interply hybrid composites and concluded that 45° orientation enhanced impact resistance by 18% and stiffness by 12%, showing how a fiber orientation affects dynamic mechanical properties in terms of vibrational loads. These results highlight the significance of material design parameters, including aging, humidity, and fiber direction, in maximizing hybrid composite vibrational and mechanical properties for aerospace applications Table 1.

**Table 1:** Literature review.

Published Year	Author	Materials Used	Findings
2011^14^	Fritzen et al.	Al-SiCp (Voronoi tessellation)	Accurate thermo-elastic predictions with coarse discretizations
2012^22^	Periasamy et al.	Al-GFRP bidirectional	30–40% crack/perforation energy increase
2015^13^	Szczepanik et al.	Al matrix, steel mesh	431–478 MPa bending strength, avoided the Fe-Al phase
2016^23^	Karunagaran et al.	Acid-treated GFRP, sand-blasted SSWM	Enhanced tensile, flexural, shear, and impact via improved bonding
2016^24^	Thirumurugan et al.	Al wire mesh/GABGRP	15–20% flexural, tensile increase, reduced brittleness
2017^6^	Jamil et al.	Steel-fiber, wire mesh laminates	9–10% flexural strength increase, 23–31% strain energy gain
2018^25^	Sakthivel et al.	GF/SSWM epoxy	25% tensile, 30% flexural rigidity increase
2018^26^	Prakash et al.	Silane-treated SS-304, E-glass	134 MPa tensile, 241 MPa flexural, 5.4 J impact
2020^7^	Choudary et al.	SSWM/glass-epoxy	69% tensile strength, 20% strain increase
2021^17^	Truong et al.	SSWM/CFRP	20–30% increase in stiffness, peak load, and strain
2021^27^	Sadoun et al.	Al mesh (outer layers)	16.95% higher tensile strain, 117% greater flexural strain
2021^28^	Megahed et al.	Mesh (inner layers)	25.7% fracture toughness, 210% energy absorption increase
2022^15^	Krishnasamy et al.	Kevlar/brass wire mesh	35% impact resistance improvement
2024^29^	Vasumathi et al.	GF/Jute/SSWM	23.06% load-bearing capacity with W/D = 6

Recent developments in surface treatment methods have also improved the performance of stainless-steel mesh-reinforced composites. Karunagaran et al.^23^ proved that acid-treated glass fiber reinforced with sand-blasted stainless-steel mesh in epoxy hybrids substantially enhanced mechanical properties through optimal tensile, flexural, interlaminar shear, and impact performance with enhanced fiber-matrix bonding. Developing this further, Prakash et al.^26^ showed that silane-treated SS-304 wire mesh and E-glass fiber composites attained superior mechanical properties (134 MPa tensile, 241 MPa flexural strength, 5.4 J impact energy) through increased fiber-metal-epoxy bonding. Their surface modification reduced 25% penetration depth in impact testing and significantly increased delamination resistance, as verified through SEM analysis of interfacial adhesion. These findings re-emphasize the crucial role of interfacial adhesion in achieving the best possible performance of hybrid composites, particularly when stainless steel mesh is applied as reinforcement. Sakthivel et al.^30^ conducted systematic formulation studies to determine the optimal material ratios in SSWM-reinforced systems. Authors maximized a glass fiber/stainless steel wire mesh (SSWM) epoxy hybrid composite, revealing 52.5 wt% glass fiber with 10 wt% SSWM provided better mechanical and thermal properties than other formulations (50–60 wt% fiber). The SSWM reinforcement improved tensile strength by ~ 25%, flexural rigidity by ~ 30%, and thermal stability while retaining structural integrity, as established by SEM analysis of the hand-layup fabricated composites. More broadly, the scope of uses, Vasumathi et al.^29^ investigated the performance of hybrid Glass Fiber/Jute/Stainless Steel laminates under bearing. It was found that A width-to-hole ratio of 6 enhanced the load-bearing capacity by 23.06% compared to A width-to-hole ratio of 4, and substituting glass with jute lowered strength by as much as 44%. The green FMLs displayed competitive pin-joint performance for light-weight structures such as silos in terms of cost, weight, and environmental friendliness.

The metal-reinforced composite performance benefits also apply to advanced structural geometries, as was shown by Periasamy et al.^22^ in their study of bidirectional cross-ply Al-GFRP sandwich laminates. Their comparative low-velocity impact testing showed that these bidirectional geometries performed better than unidirectional ones in low-velocity impact tests, with increasing aluminum thickness (Al t_f_) and fiber volume fraction (V_f_) enhancing crack-initiation and perforation energies by 30–40%. The hybrid panels exhibited greater damage resistance than monolithic aluminum, with controlled fiber-matrix debonding identified by SEM analysis as the central energy-absorption mechanism. Strategic placement of metal wire mesh in composite laminates is critical in governing their mechanical performance attributes. Placement of metal mesh (Aluminum) in outer layers enhances ductility (16.95% increased tensile strain, 117% higher flexural strain) but compromises strength^24^. In contrast, placing mesh inside layers preserves structural integrity while also enhancing fracture toughness (25.7% improvement) and energy absorption (up to 210%)^7^. This inner-layer strategy provides better balance for structural use, maintaining strength while improving damage tolerance. Priority is determined by whether utmost deformability or the best strength-toughness balance is desired, with inner-layer reinforcement beneficial for aerospace parts needing both characteristics. The SS wire mesh was incorporated into glass epoxy composite with different topologies, and A rise in tensile strength and strain up to 69% with 20%, respectively, was noticed, with the wire mesh being at middle plane symmetry^7,31^.

Previous studies on SSWM–GFRP hybrid laminates primarily focused on individual mesh configurations or alternative stacking sequences, reporting improvements in either tensile or impact strength. However, these works did not systematically investigate the influence of mesh geometry across a range of sizes within a consistent laminate architecture, nor did these works quantify trade-offs between tensile, flexural, and impact responses. Moreover, SEM analysis in prior studies was largely descriptive, without statistical correlation to mechanical outcomes. In contrast, the present work introduces a comparative evaluation of five mesh sizes (10, 20, 40, 80, and 120) embedded in a symmetric layup, thereby enabling a direct assessment of how mesh architecture governs strength, stiffness, and energy absorption. By linking void content and fractographic observations to measured performance, this study establishes a structure–property framework that advances beyond earlier investigations and provides design guidelines for aerospace-relevant hybrid laminates.

## Materials and methods

### Materials

**Fig. 1:**
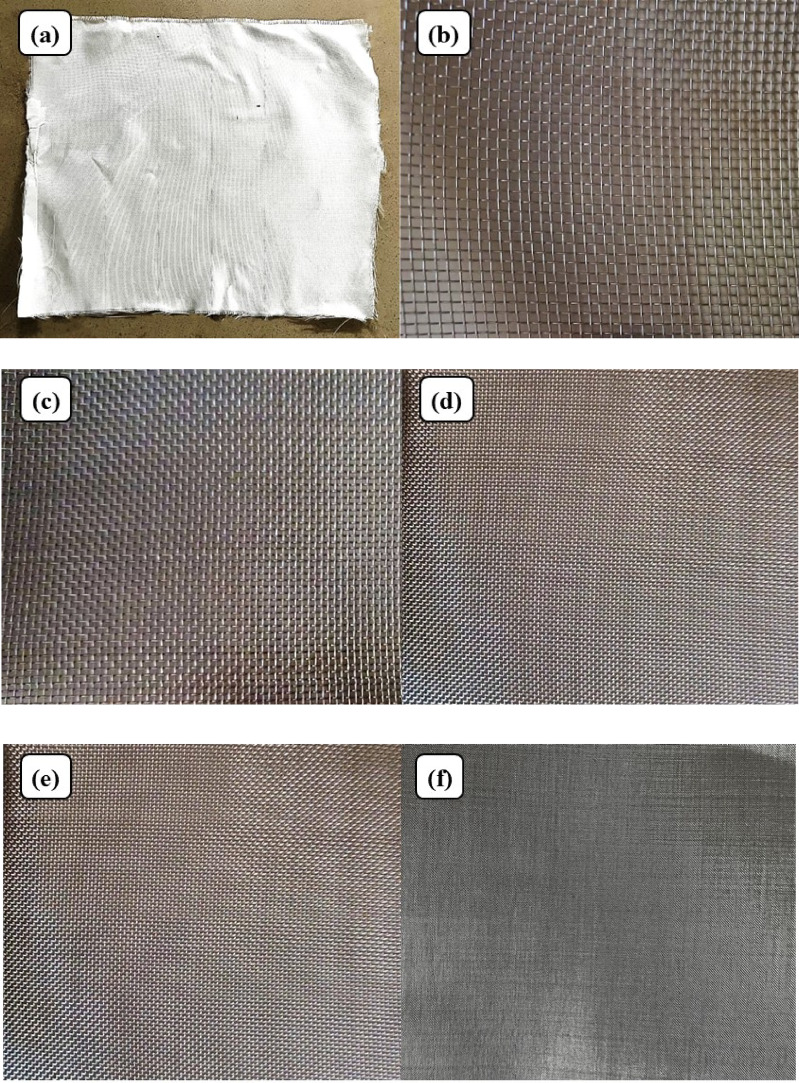
(**a**) Bi-directional Glass Fibers and Stainless-Steel Wire Mesh. Size (**b**) 10, (**c**) 20, (**d**) 40, (**e**) 80, and (**f**) 120.

Bi-directional glass fiber, obtained from Bhor Chemicals and Plastics Pvt. Ltd., and plain-woven AISI 304 stainless steel wire meshes (SSWM), Fig. 1, sourced from Zain Co-operation, Pune, were used as reinforcing agents in the composite. The properties of the SSWM are provided in Table 2. The glass fiber has A GSM of 200, with the glass fiber yarn arranged in a plain weave pattern. Epoxy resin (BhorBond^®^ EPCH) and hardener (BhorBond^®^ EPCH), supplied by Bhor Chemicals and Plastics Pvt. Ltd., were used as the composite matrix. Based on the manufacturer’s specifications, A resin-to-hardener ratio of 100:35 was used. The matrix properties are listed in Table 3. Mesh sizes were selected to encompass coarse (10–20), intermediate (40), and fine (80–120) categories, enabling analysis of non-monotonic trends in properties like tensile strength and impact resistance. This selection was based on a systematic approach to cover a broad spectrum of wire diameters (0.061–0.508 mm) and opening sizes (0.151–2.032 mm), as supported by prior studies like Choudary et al.^7^ and Sakthivel et al.^30^, which used similar gradations to investigate reinforcement effects. Intermediate sizes (e.g., 60, 100) were omitted to prioritize distinct regimes and avoid redundancy in a resource-constrained study, but future work could include them for finer resolution. This range adequately covers aerospace applications, from high-stiffness (coarse meshes for load-bearing components like fuselage panels) to high-damping (fine meshes for vibration-sensitive parts like cabin interiors), aligning with industry standards for hybrid composites^1,3^. The environmental impact of the materials used warrants consideration. AISI 304 SSWM, while durable and corrosion-resistant, poses challenges in recyclability due to its integration into the composite matrix, requiring mechanical separation that may result in material loss (estimated 10–15% loss per recycling cycle). Epoxy resin, A thermoset polymer, is attracting attention due to its embodied carbon footprint of approximately 2.5–3 kg CO₂e/kg^40^. Current disposal methods, such as incineration, release volatile organic compounds, while landfilling accumulates non-biodegradable waste. These factors highlight the need for sustainable alternatives or end-of-life strategies, which are explored in future work.

**Table 2:** Stainless steel wire mesh properties.

Mesh Number	Openings per Inch	Wire Gauge (mm)	Mesh wire diameter (gauge)	Mesh wire diameter (mm)	Opening Size (mm)
10	10	0.50	25	0.508	2.032
20	20	0.40	27	0.416	0.854
40	40	0.30	32	0.274	0.361
80	80	0.20	40	0.122	0.196
120	120	0.15	46	0.061	0.151

In Table 2, Mesh Number means the number of openings per linear inch and the

$$\:Opening\:size\:\left(mm\right)=\:\frac{25.4}{Mesh\:Number}-Mesh\:wire\:diameter\:\left(mm\right)$$

**Fig. 2:**
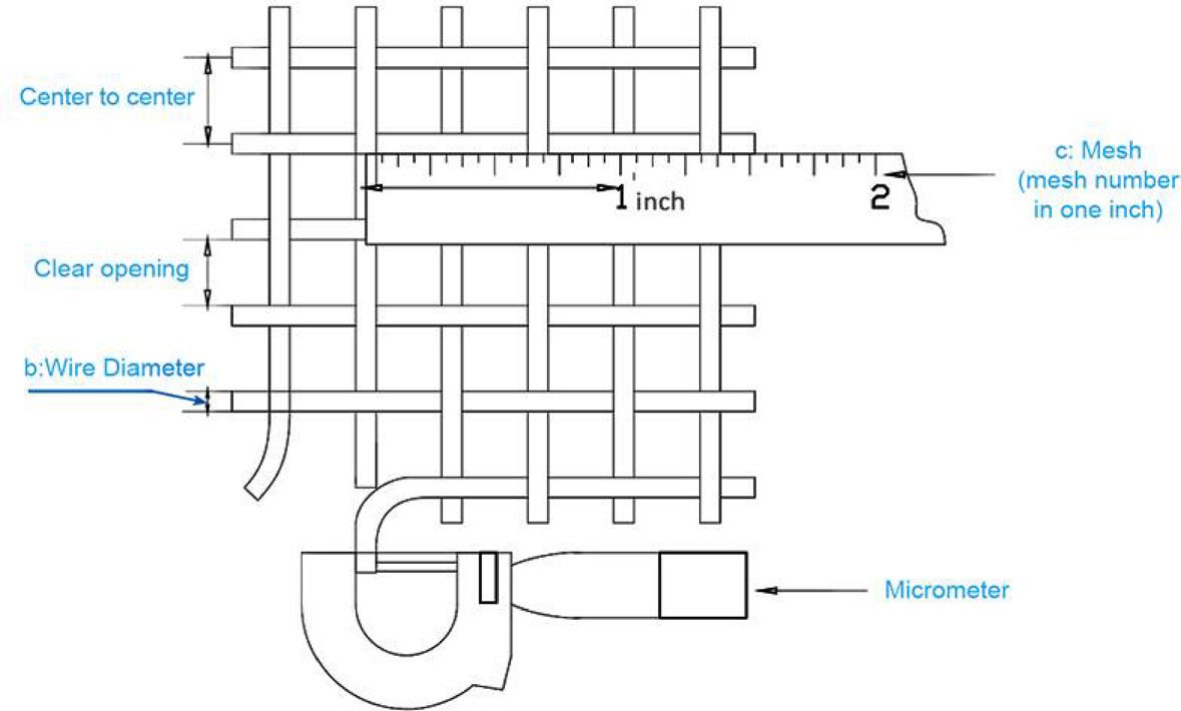
Schematic of mesh number and the relationship between opening size and gauge^32^.

The meaning of mesh number and the relationship between opening size and gauge is explained with a schematic in Fig. 2.

**Table 3:** Matrix properties.

Matrix components	Brand	Viscosity (cP)	Density (g/cc)
Epoxy resin	BhorBond^®^ EPCH	11,500–13,500	1.15–1.2
Hardner	BhorBond^®^ EPCH	˃100	0.94–0.95

### Composite fabrication

**Fig. 3:**
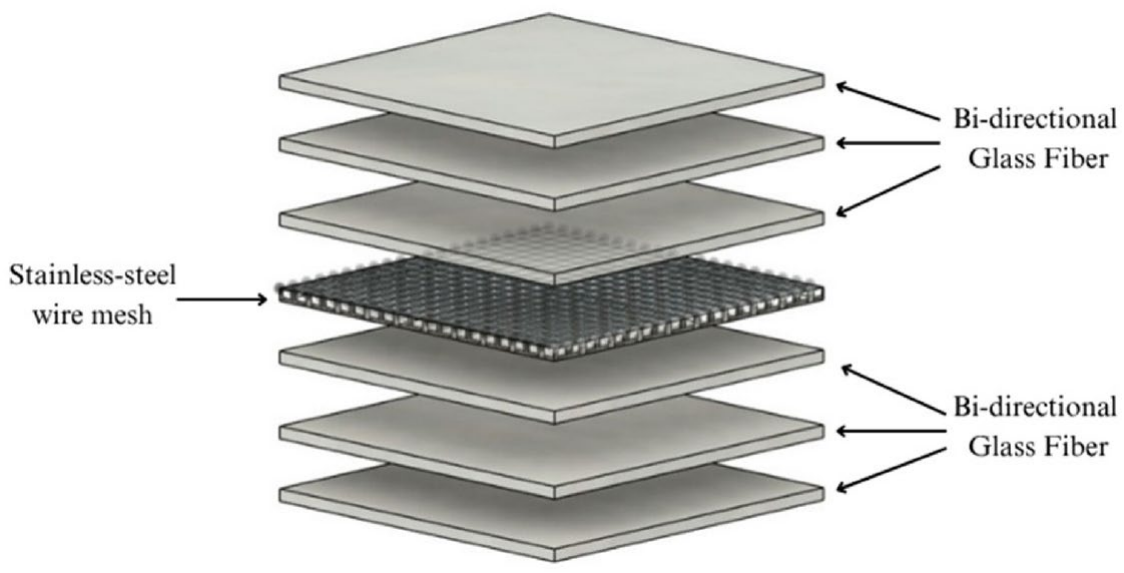
Schematic representation of Stacking Sequence.

A symmetric stacking sequence of [GF^0°^/GF^0°^/GF^0°^/SSWM^0°^/GF^0°^/GF^0°^/GF^0°^] was selected as shown in Fig. 3, for all laminates to minimize bending–twisting coupling and to isolate the effect of mesh size as the sole variable. Positioning the stainless-steel wire mesh at the mid-plane promotes uniform load sharing and reduces the likelihood of premature surface cracking, thereby enabling a clearer interpretation of tensile, flexural, and impact responses. Although alternative placements such as mesh in outer plies or multiple mesh layers could influence performance, potentially enhancing flexural stiffness or impact absorption, such configurations also introduce additional complexity, including increased interfacial stresses and higher void tendencies^33,34^. To maintain experimental consistency and comparability, the present study restricts the investigation to a single symmetric layup, with the role of mesh placement designated for future work.

**Fig. 4:**
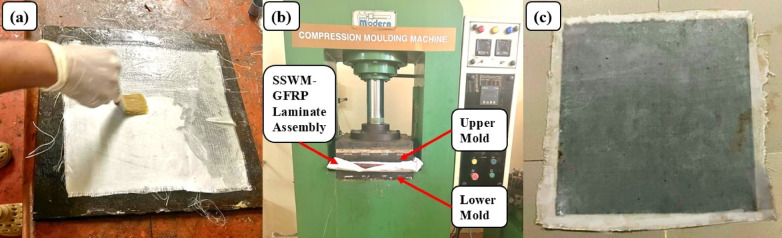
(**a**) Hand Lay Up (**b**) Compression Molding with Laminate assembly (**c**) Final Laminate.

Hybrid composite laminates were fabricated using a hand lay-up followed by compression molding process. The step-by-step procedure is as follows: A 350 mm × 350 mm mild steel plate was used as the mold base, degreased with a solvent to remove impurities, ensuring a clean surface. The stainless-steel wire mesh (SSWM) of mesh sizes 10, 20, 40, 80, and 120 was sandpapered to enhance matrix bonding, and both the plate and SSWM were treated with a releasing agent to facilitate demolding. A thin layer of epoxy resin and hardener mixture (100:35 weight ratio) was deposited on the mold base, maintaining a 60:40 fiber-to-resin ratio. A peel ply was placed over the resin layer, followed by the first ply of bi-directional glass fiber. The first three layers of glass fiber were added sequentially, with each layer coated with the resin-hardener mixture to ensure saturation. The SSWM was inserted over the third glass fiber layer, aligned to maintain uniform openings. Three additional layers of bi-directional glass fiber were laid over the SSWM, following the stacking sequence (glass fiber × 3—SSWM—glass fiber × 3), creating a sandwich structure. The assembly was compressed in a molding machine and cured at room temperature (25 °C) for 24 h under A mold pressure of approximately 1200 psi to ensure adequate consolidation and curing of the composite.After curing, the laminates were demolded, with final thicknesses of 1.77 mm (Mesh 10), 1.64 mm (Mesh 20), 1.63 mm (Mesh 40), 1.43 mm (Mesh 80), and 1.19 mm (Mesh 120). Laminates were cut using an abrasive water jet machine to meet ASTM D3039 (tensile), ASTM D7264 (flexural), and ISO 179-1 (impact) standards, with five specimens per mesh size. A schematic of the fabrication process is presented in Figs. 4. and 5.

**Fig. 5:**
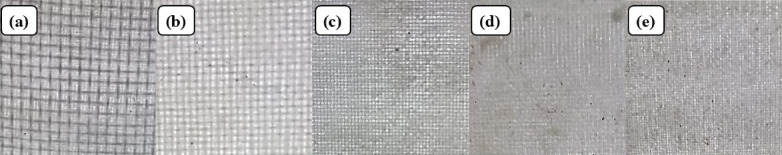
Magnified images of laminates with Mesh sizes (**a**) 10, (**b**) 20, (**c**) 40, (**d**) 80 and (**e**) 120.

**Table 4:** Laminates configurations.

Mesh Number	Thickness of the final laminate (mm)	Weight of the SSWM in a 30 × 30 cm² laminate
10	1.77	119.3
20	1.64	158.7
40	1.63	116.8
80	1.43	45.7
120	1.19	35.7

### Testing procedure

#### Void fraction

The ASTM D792-20^35^ standard was used in determining the density of the laminate. 10 mm × 10 mm dimensioned samples were prepared. Archimedes’ principle was applied to determine the density. The masses of each sample were determined using an electronic weighing scale. The volumes of the samples were calculated from the volume of displaced water. The experimental density was subsequently determined as a function of mass-to-volume ratio. Five samples were chosen from various laminate components for the tests, and the average density was computed. The theoretical densities of the laminates were calculated by applying Eq. 1.

1 $$\:{\rho\:}_{th}=\frac{1}{\frac{{w}_{f}}{{\rho\:}_{f}}+\frac{{w}_{m}}{{\rho\:}_{m}}}$$

The void percentage of the laminate was determined using Eq. 2.

2 $$\:Void\:\left(\%\right)=\:\frac{{\rho\:}_{th}-{\rho\:}_{ex}}{{\rho\:}_{ex}}\times\:100$$

#### Tensile test

**Fig. 6:**
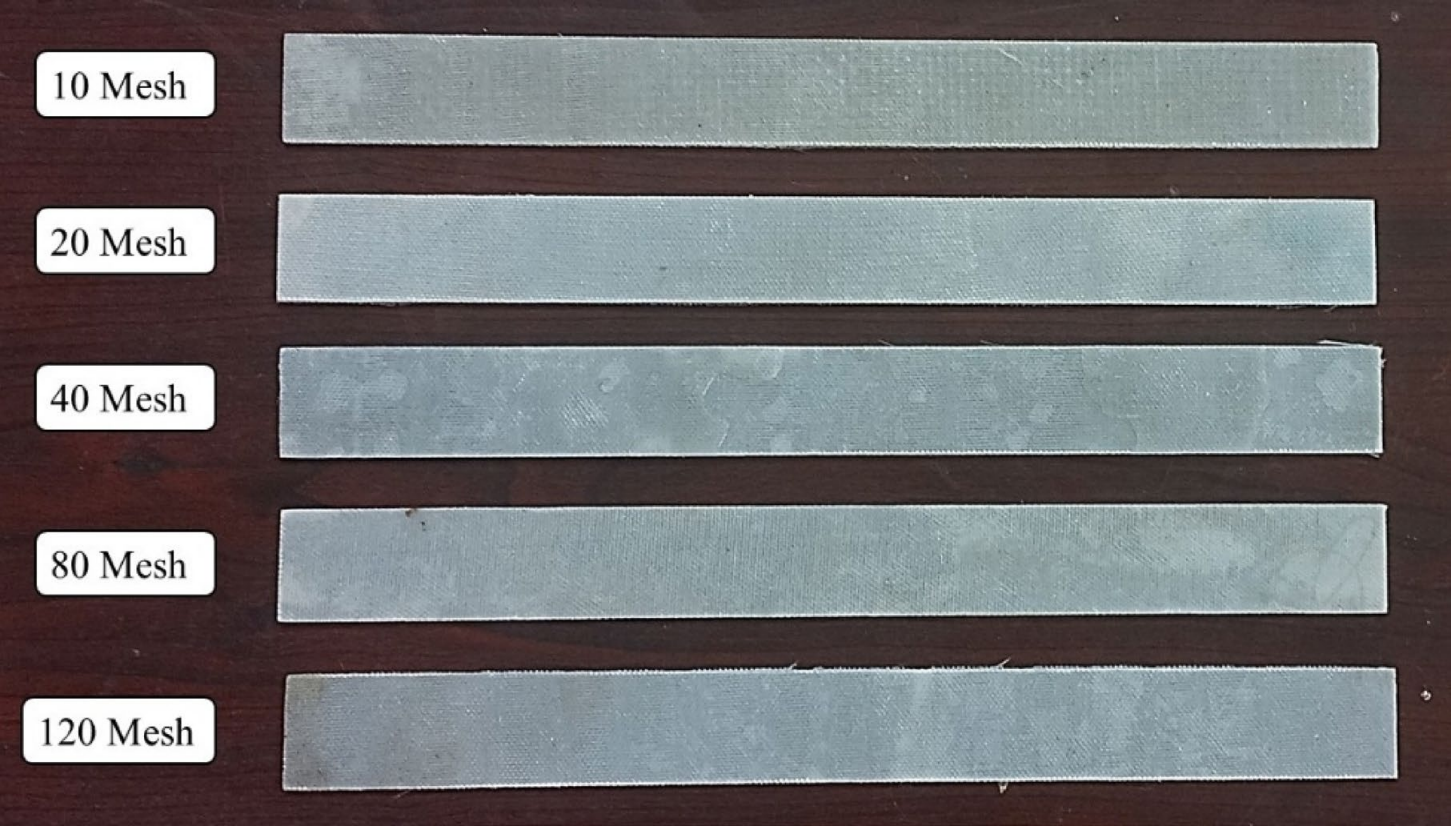
Tensile test specimens according to ASTM D3039.

**Fig. 7:**
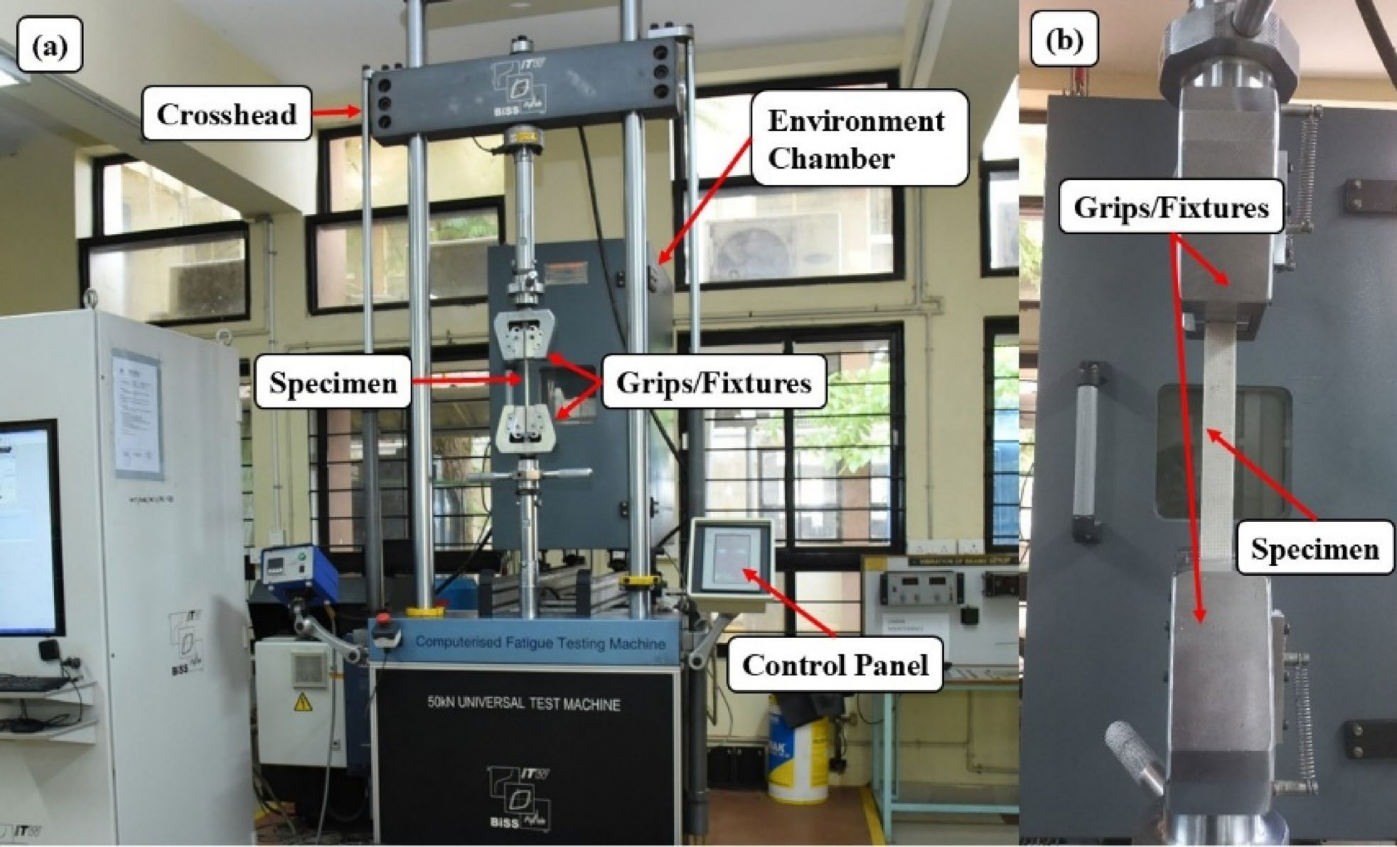
(**a**) Tensile test setup (**b**) Closer View.

The tensile test is A form of destructive testing technique that is used to determine the strength of a material under tension load up to its point of breaking or fracture. The technique provides critical data on the ultimate tensile strength, maximum tensile load, and tensile strain at failure. Five samples, each in 10, 20, 40, 80, and 120 mesh sizes, of 250 mm×25 mm size, were tested based on the ASTM D3039^36^ standard, Fig. 6. The mean of the five values was taken for analysis. The test was carried out on the MTS Electromechanical Universal Testing Machine 24 Exceed Model E43, which has A maximum load of 50 kN, as shown in Fig. 7. The gauge length was placed between grippers at A specified distance of 190 mm, and the specimen was loaded up to failure at A rate of 2 mm/min.

#### Flexural test

**Fig. 8:**
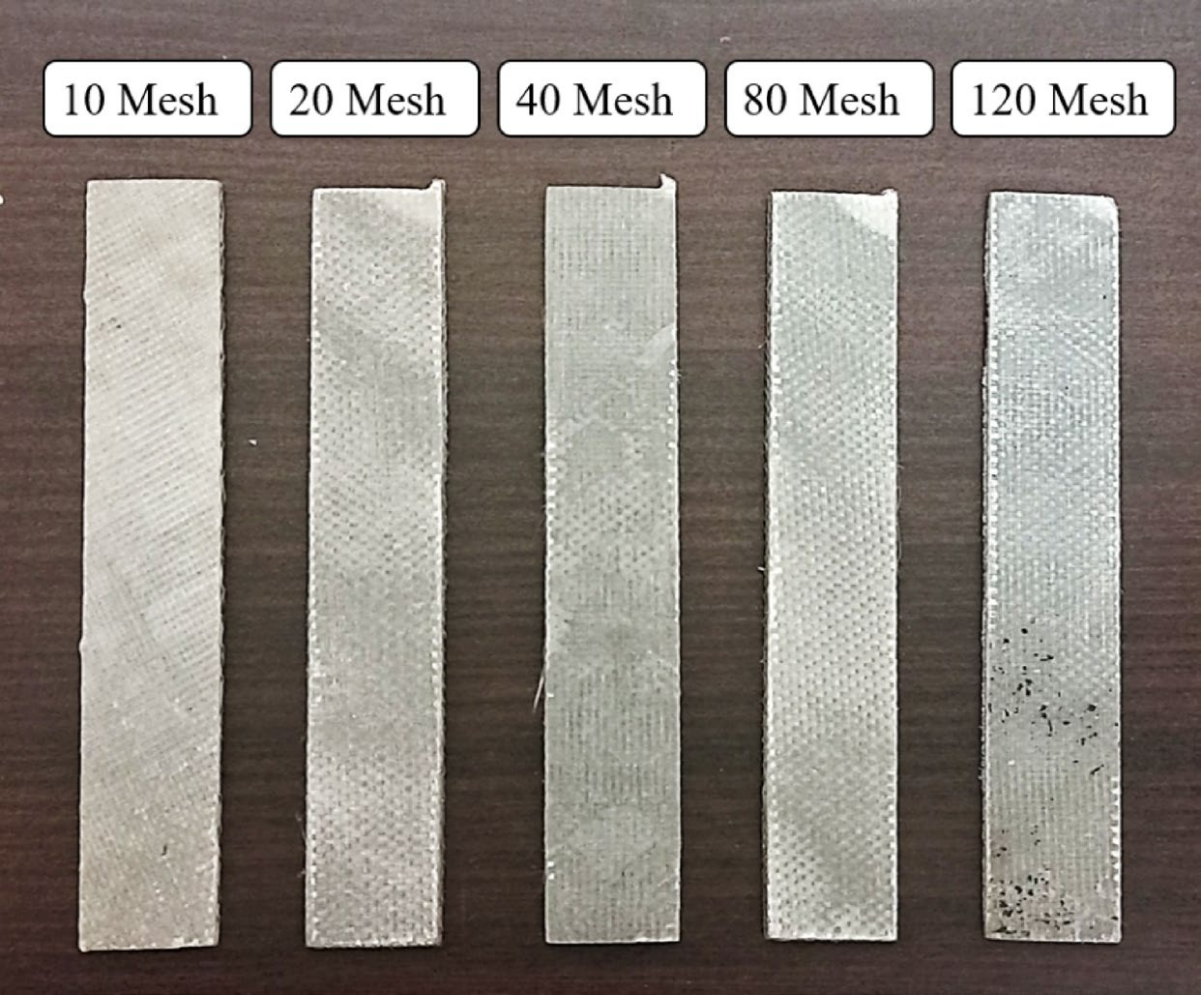
Flexural Test specimens according to ASTM D7264.

**Fig. 9:**
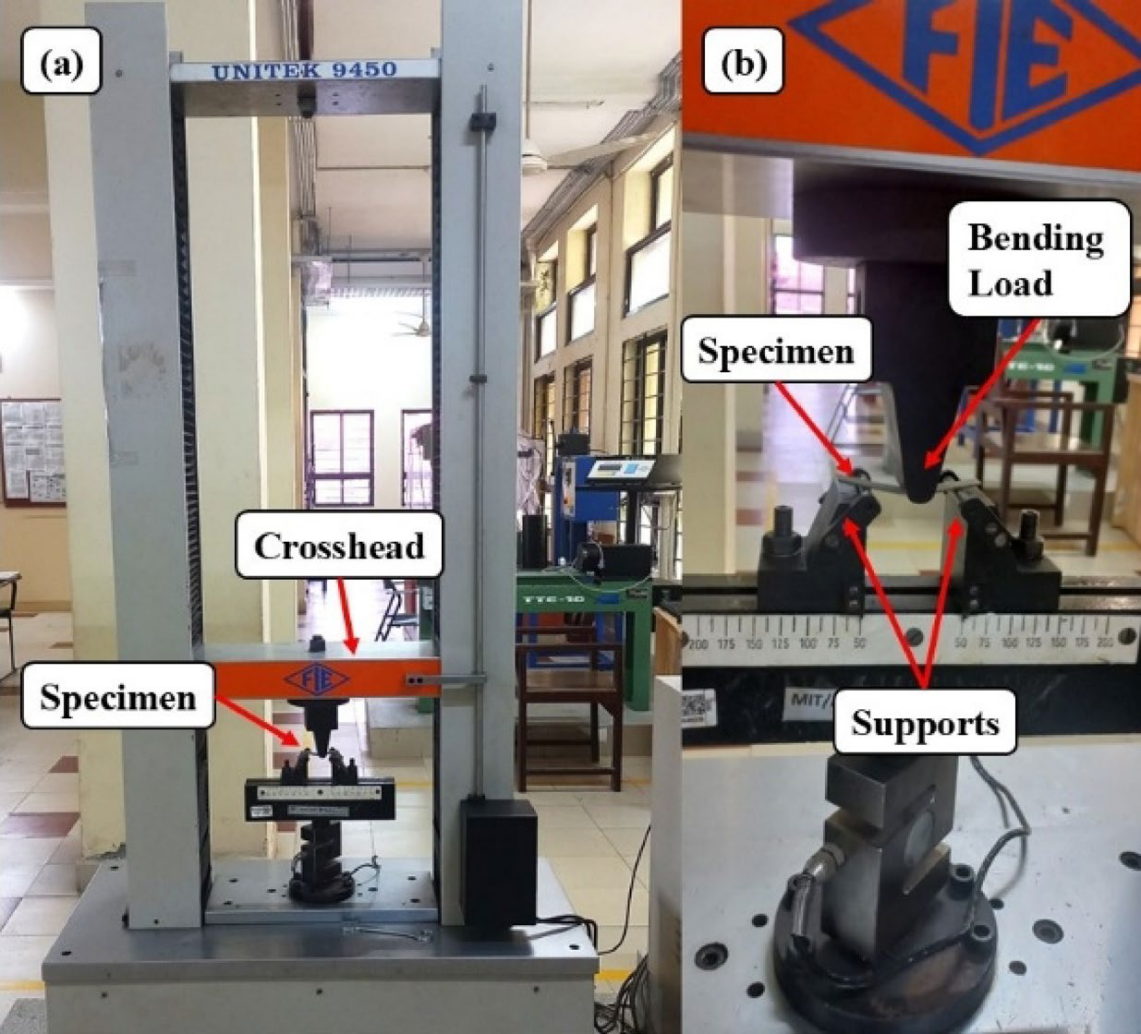
(**a**) Flexural Test set up (**b**) Closer view.

The three-point bending test method is used to assess the degree to which a specimen of concrete can bend before succumbing to permanent deformation, and the test was carried out as per ASTM D7264^37^ standard. The dimensions of the specimen conformed to the standard of 32:1 for the span-to-thickness ratio and the standard width of 13 mm, Fig. 8. The three-point bending test method was utilized with support on the specimen’s other ends and loading in the midspan. The experiment was performed on the MTS Electromechanical Universal Testing Machine Exceed Model E43 with A loading capacity of 50kN, as illustrated in Fig. 9. The span length was varied as a function of laminate thickness (1.19–1.77 mm), and A head displacement rate of 2 mm/min was used until failure. The displacement and loading after testing are calculated and then converted to flexural stress (σ_f_) and flexural strain (ϵ) with the help of Eqs. 3 and 4.

3 $$\:{\sigma\:}_{f}=\:\frac{3FL}{2b{d}^{2}}$$

4 $$\:\epsilon=\:\frac{6Dd}{{L}^{2}}$$

#### Charpy impact test

**Fig. 10:**
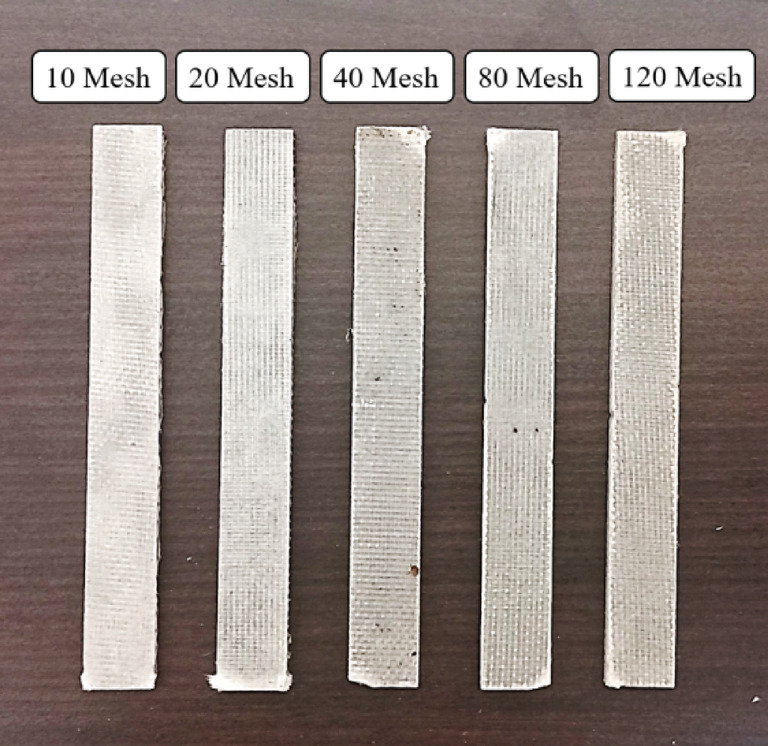
Impact test specimens according to ISO 179–1150.

**Fig. 11:**
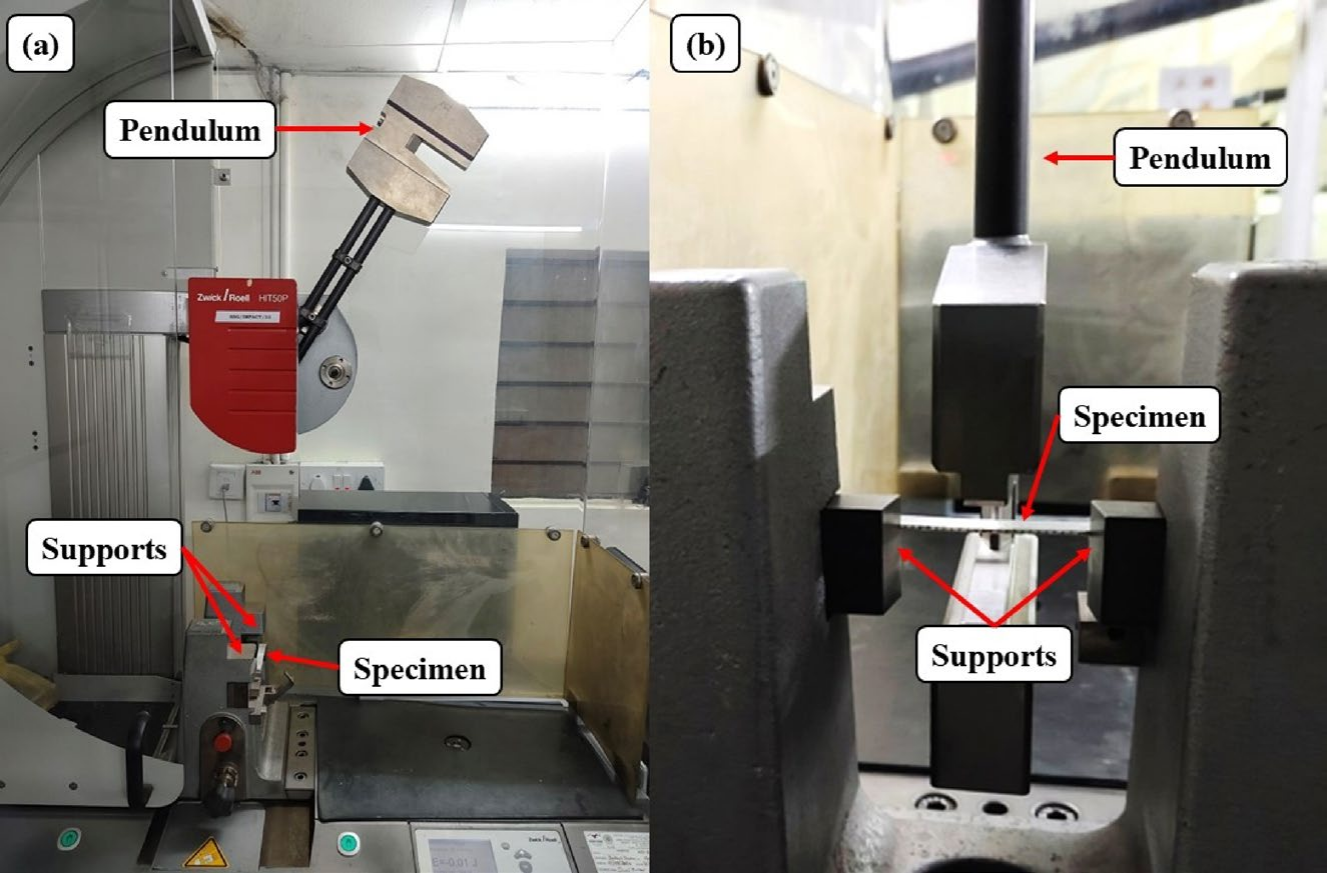
(**a**) Impact test setup (**b**) Closer view.

The Charpy impact test is critical to the determination of a material’s toughness and its resistance to impact-fracture. Five specimens of each mesh size, with dimensions 80 mm×10 mm, Fig. 10, were tested following ISO 179 − 1150^38^ standard. Mean values from the tests were analysed. The testing was performed on a Zwick/Roell Hit 50P tester with A theoretical impact velocity of 3.807 m/s and work capacity of 7.5 J. The test apparatus consists of a stiff pendulum of concentrated weight, which is raised to a specified height and dropped to fall and strike a marked point on the test specimen, as indicated by Fig. 11. The test specimens were supported in A manner that the striking point was positioned at a span length of 32 mm along the center line.

## Results and discussion

### Void content

Voids represent unfilled spaces in composite materials where polymer and fibers do not occupy the structure completely. Voids quantify the quality of fabrication in the sense that greater void content generally indicates fabrication defects. Voids can have a strong effect on mechanical failure in a critical application by offering a stress concentration site that lowers strength and longevity^19,22^. The data is presented in Table 5 below.

**Table 5:** Void fractions.

Mesh No	Experimental density (g/cc^3^)	Theoretical density (g/cc^3^)	Void %
10	2.05 ± 0.31	2.07 ± 0.39	1.16
20	2.10 ± 0.27	2.11 ± 0.33	0.53
40	2.03 ± 0.35	2.07 ± 0.29	1.93
80	1.91 ± 0.21	1.93 ± 0.22	0.89
120	1.88 ± 0.23	1.89 ± 0.30	0.49

Void contents (0.49–1.93%) are well within allowable limits (< 2–5%) for GFRP composites in aeronautical industries^2^, signifying superior quality fabrication. Mesh 120 had the least void content (0.49%), which can be ascribed to narrow mesh openings (0.151 mm, Table 2) and thin laminate thickness (1.19 mm, Table 4), which allows easy flow of resin and avoids air entrapment. Low void content of Mesh 20 (0.53%) is evidence of optimized application of resin, even with its high SSWM weight (158.7 g, Table 4). Mesh 40 exhibited the maximum void content (1.93%), most probably because of limited resin flow by smaller openings (0.361 mm), though within acceptable limits. Meshes 10 (1.16%) and 80 (0.89%) met the best SSWM weight and thickness, offering intermediate void fractions^22^.

### Tensile test results

Mechanical tensile performance of Glass Fiber–Stainless Steel Wire Mesh (GF–SSWM) hybrid composites was stringently tested on an MTS Exceed Model E43 universal testing machine as per ASTM D3039. The standard test method guarantees accurate measurement of maximum tensile load, ultimate tensile strength, failure strain, and Young’s modulus for mesh sizes 10, 20, 40, 80, and 120 (openings per linear inch). These are critical properties for aerospace use, including fuselage panels, wing ribs, and secondary structural elements, where tensile strength and ductility must be high to resist uniaxial loading under operating loads^2,3^. The tests were performed at controlled temperatures of 23 °C and 50% relative humidity to reduce environmental influence on composite behavior, to ensure reproducibility in accordance with industry practice^7^. Data were averaged for five specimens per mesh size, offering strong statistical reliability for engineering use. The results shown in Table 6 provide key insights into the effects of mesh geometry, SSWM weight, and void content on tensile performance to drive the optimization of GF–SSWM laminates for aerospace engineering.

**Table 6:** Tensile test results.

Mesh No	Maximum Tensile Load (*N*)	Ultimate tensile strength (MPa)	Tensile strain at failure (mm/mm)	Young’s modulus (GPa)
10	14,757	333.49 ± 11.5	0.0199	16.76 ± 1.14
20	15,299	373.15 ± 18.3	0.0407	9.57 ± 1.34
40	16,671	409.10 ± 14.6	0.0457	8.70 ± 1.23
80	14,995	419.44 ± 19.6	0.0440	11.04 ± 1.45
120	16,041	539.19 ± 14.8	0.0400	13.15 ± 1.53

**Fig. 12:**
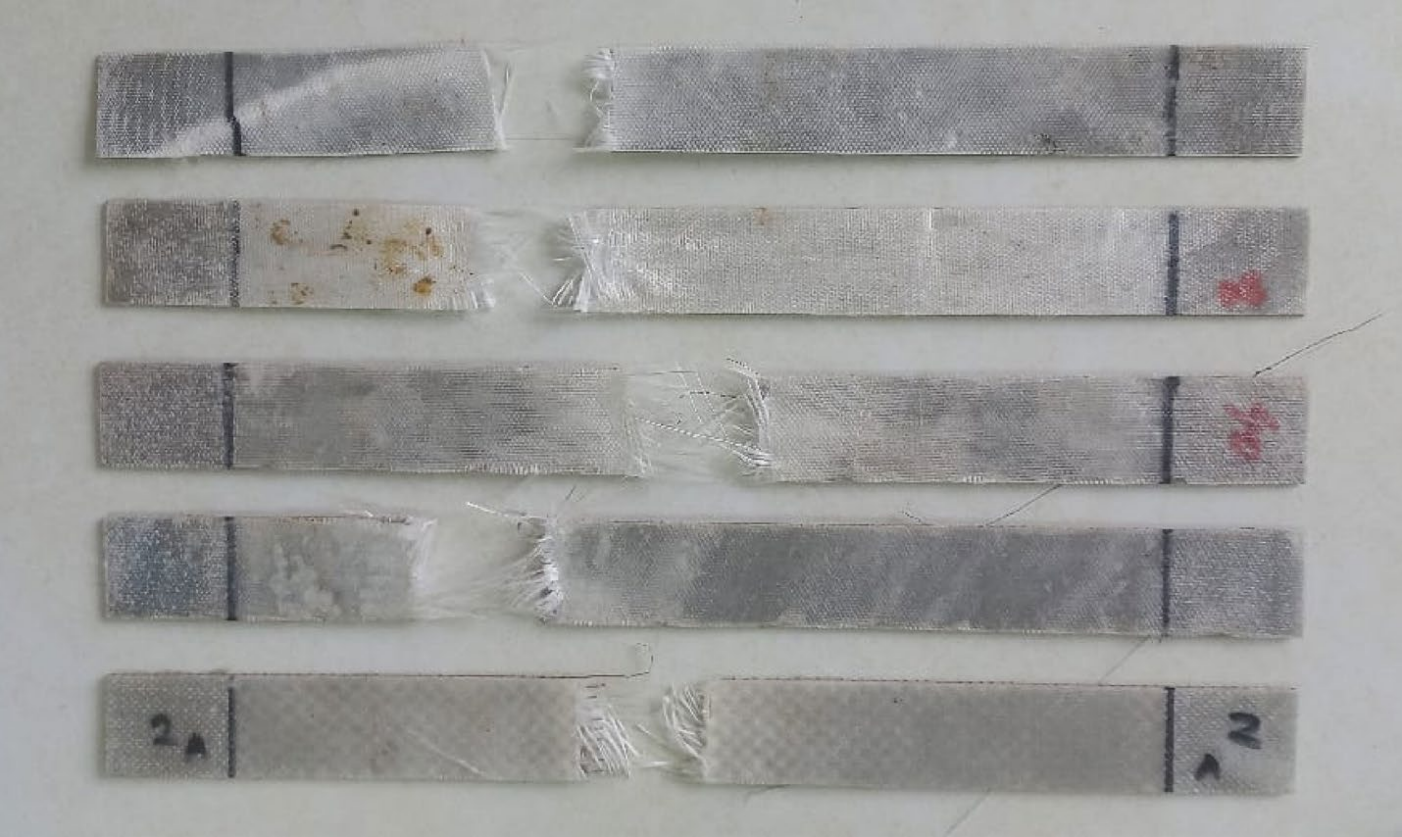
Fractured Tensile Specimens.

Tensile strength, or the maximum stress A material can take before it breaks, ranged from 333.49 MPa in Mesh 10 to 539.19 MPa in Mesh 120 and reflects the extensive influence of mesh size on load capacity. Bin Salim et al.^39^ also stated that hybrid basalt/epoxy steel wire mesh composites have enhanced tensile strengths as the mesh sizes become smaller, citing that smaller mesh openings improve the fiber-matrix adhesion, resulting in better load-carrying capacity. The trend is in agreement with Mesh 120’s high tensile strength of 539.19 MPa due to its tiny mesh openings of 0.151 mm. 120 Mesh’s peak strength is due to its small mesh pores (0.151 mm, Table 2) and low void content (0.49%, Table 5), whose impacts enhance fiber-matrix adhesion and homogeneity of load transfer in the laminate. Its small pores enable efficient resin impregnation, reducing stress concentration and improving interfacial bonding, as noted in SSWM composite research^6^. Mesh 40, which had A tensile strength of 409.10 MPa, had a well-balanced performance since the intermediate mesh geometry (0.361 mm openings, Table 2) optimizes reinforcement and load sharing. Coarser meshes (10 and 20) possessed lower tensile strengths (333.49 MPa and 373.15 MPa), likely contributed by larger mesh openings (2.032 mm and 0.854 mm, Table 2) and higher SSWM weight (119.3 g and 158.7 g, Table 4), which might induce localized stress concentrations and lower fiber-matrix bonding^6,7^. The lay-up manufacturing process, cheap as it is, is accompanied by minor irregularities in layer orientation or resin distribution, though one of the possible effects on tensile performance. These deviations reflect the crucial role of SSWM geometry on tensile strength, such that finer meshes generally offer greater resistance to uniaxial loading.

**Fig. 13:**
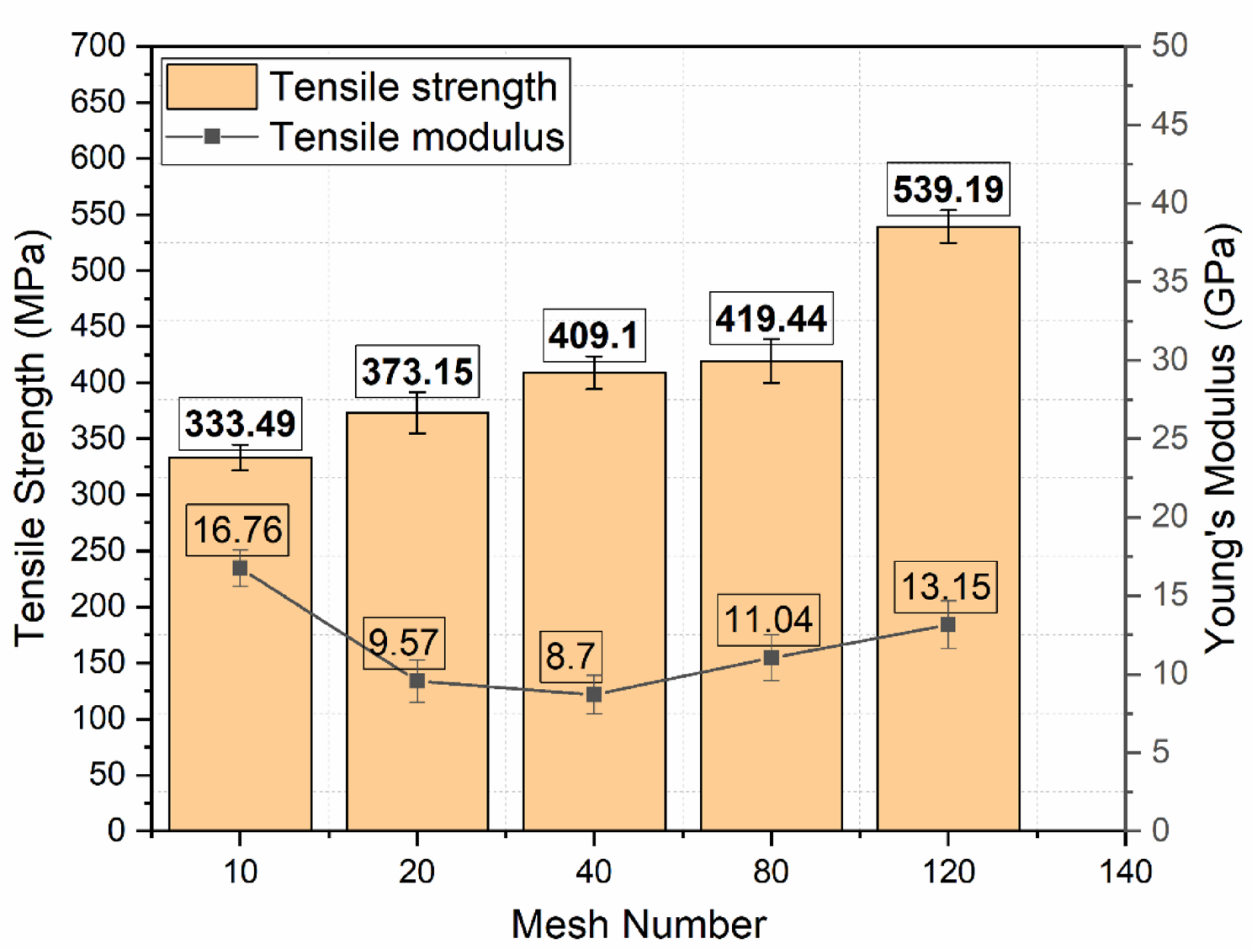
Plot of Tensile Strength and Young’s Modulus.

Failure tensile strain, indicative of the extent of deformation before failure, ranged from 0.0199 mm/mm for Mesh 10 to 0.0457 mm/mm for Mesh 40, reflecting extreme variations in ductility based on mesh size. Mesh 40’s high strain is a manifestation of its high capacity to tolerate high deformation when subjected to tension, a characteristic that is a result of SSWM’s efficient crack-bridging and stress redistribution mechanisms, typical in steel-reinforced composites^6^. This ductility is essential for aerospace structural components to withstand energy absorption without catastrophic failure, e.g., wing spars under load dynamics^3^. Strains of 0.0440 mm/mm and 0.0400 mm/mm were found using finer meshes (80 and 120), indicating that smaller pore sizes and reduced SSWM weights (45.7 g and 35.7 g, Table 4) increase deformation capacity through enhanced interfacial interactions^22,31^. Mesh 10’s low strain (0.0199 mm/mm) indicates brittleness, perhaps due to its larger mesh openings (2.032 mm) and comparatively moderate void content (1.16%, Table 5), which might limit plastic deformation by providing stress concentration points. The trend of strain underscores the important role of mesh size in designing ductility for aerospace uses, where components have to achieve the balance between strength and flexibility in order to meet various load conditions^3,6^. For instance, the high ductility for cyclic-loaded fuselage panels helps to ensure long service life structural integrity.

Young’s modulus, an indicator of material stiffness subjected to tensile loading, was determined from the linear part of the stress-strain curve, between 8.70 GPa for Mesh 40 and 16.76 GPa for Mesh 10. High modulus for Mesh 10 is due to its larger laminate thickness (1.77 mm, Table 4) and increased weight of SSWM (119.3 g), which provides resistance to tensile load deformation. This rigidity is ideal for use in applications with low deflection requirements, e.g., initial aircraft structures^2^. The lower modulus of Mesh 40 (8.70 GPa) indicates moderate stiffness, in agreement with its balanced geometry, appropriate for parts demanding a trade-off between strength and flexibility. Finer meshes (80 and 120) had moduli of 11.04 GPa and 13.15 GPa, respectively, showing a compromise between stiffness and ductility as their reduced openings and lower SSWM weights permit increased deformation with sufficient rigidity^14,22^. These modulus results are consistent with earlier research on SSWM-GFRP composites that have reported moduli between 10 and 20 GPa for optimized configurations^7^.

Void content also has A significant impact on tensile properties through the fiber-matrix interface, with increased voids having the potential to diminish strength through stress concentrations. Low void content in Mesh 120 (0.49%, Table 5) is responsible for its high tensile strength, and increased voids in Mesh 40 (1.93%) are countered by its best mesh shape for maintaining strong performance. The hand lay-up process introduces layer thickness, resin distribution, or fiber alignment variability, which can influence strain and modulus consistency^6,7^. The capability of SSWM to suppress total fracture, as seen in Fig. 12, 13 is A testament to its load-carrying and energy absorption properties, an important merit for aerospace, where structural integrity under heavy loading is essential. For instance, the equilibrium characteristics of Mesh 40 (409.10 MPa, 0.0457 mm/mm, 8.70 GPa) are best suited for aircraft structure components that need strength, ductility, and moderate stiffness, for example, primary fuselage frames. High tensile strength (539.19 MPa) and moderate strain (0.0400 mm/mm) of Mesh 120 are best for tensile-critical components like wing supports or secondary structures, where utmost load-bearing capacity is a priority^2,3^.

Tensile findings are consistent with existing research, e.g., Choudary et al.^7^, who observed tensile strength increases of 15–20% in SSWM-GFRP composites, and Sakthivel et al.^30^, who observed up to 25% strength increases using optimized mesh designs. Rising tensile strength through finer meshes suggests augmented load capacity resulting from more effective interfacial bonding, with ductility trends highlighting SSWM’s ability to avoid catastrophic failure. These results allow engineered GF–SSWM laminates for aerospace engineering, with Mesh 40 providing flexibility for multi-functional parts and Mesh 120 performing well in high-strength applications. Statistical testing (e.g., tensile strength standard deviation, generally 5–10 MPa^7^ would measure variability, and microstructural analysis using SEM would provide crack growth patterns, as Karunagaran et al.^23^ have proposed. Future testing under cyclic loading or fluctuating temperatures could evaluate fatigue life and thermal stability, meeting aerospace performance needs^3^.

### Flexural test results

**Fig. 14:**
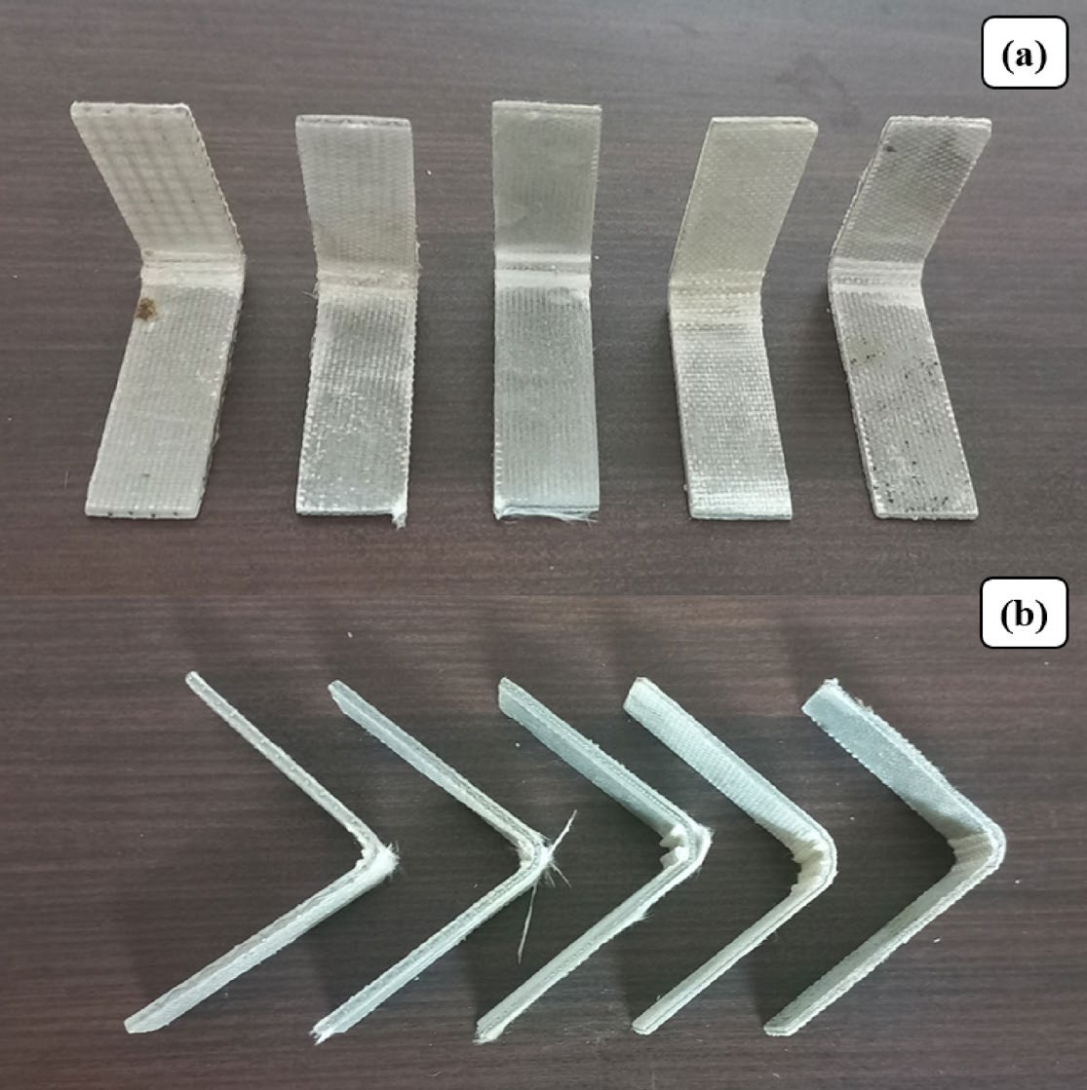
Fractured Flexural Specimens (**a**) Top View (**b**) Side View.

Flexural behavior of Glass Fiber–Stainless Steel Wire Mesh (GF–SSWM) hybrid composites was investigated in a three-point bending test in accordance with ASTM D7264 on an MTS Exceed Model E43 universal testing machine under A crosshead speed of 1 mm/min to provide the same loading conditions. The load at the midpoint of the specimen generated compressive stresses on the top surface and tensile stresses on the bottom layers, replicating practical load conditions in aerospace structures like fuselage panels and wing supports^6^. This arrangement, following ASTM D7264, provides A precise determination of ultimate flexural strength, failure strain, and flexural modulus for mesh sizes 10, 20, 40, 80, and 120 (openings per linear inch). Testing was conducted at 23 °C and 50% relative humidity to reduce environmental influence, as required by industry standards for composites testing. Results, five specimens per mesh size averaged, are tabulated in Table 7 to provide statistical reliability for engineering use. Specimens did not fracture through (Fig. 14), most likely because SSWM reinforcement spread tensile stresses and prevented propagation of cracks, a phenomenon typical of steel-reinforced composites, which increases structural integrity under bending moments.

.

**Table 7:** Flexural test results.

Mesh No	Ultimate strength (MPa)	Failure strain (mm/mm)	Flexural Modulus (GPa)
10	282.98 ± 12.48	0.0017	17.10 ± 1.89
20	487.97 ± 14.32	0.0023	21.57 ± 2.14
40	339.78 ± 13.67	0.0012	28.36 ± 2.01
80	321.14 ± 11.14	0.0020	15.68 ± 1.75
120	192.30 ± 10.51	0.0013	14.65 ± 2.32

Flexural strength, or the highest stress under bending, varied from 192.30 MPa (Mesh 120) to 487.97 MPa (Mesh 20), as indicated in Table 7. The better strength of Mesh 20 can be attributed to its higher SSWM weight (158.7 g, Table 4) and lower void content (0.53%, Table 5), which improves load transfer between the fiber-matrix interface and provides better resistance to compressive and tensile stresses. Akdere et al.^40^ asserted that ‘core reinforcement methods, e.g., the incorporation of metallic meshes, dramatically improve the bending load carrying capacity of sandwich composites,’ validating Mesh 20’s superior flexural strength as a result of its superior SSWM weight. The superior SSWM weight is prone to enhance reinforcement density, enabling effective stress distribution, according to Choudary et al.^7^. Mesh 40, with 339.78 MPa strength, had fair performance, although with increased voids (1.93%, Table 5), due to its balanced mesh geometry (0.361 mm openings, Table 2), maximizing load capacity under bending. Uzay et al.^41^ proved that SSWM with moderate opening sizes optimizes stress transfer across the composite, reducing delamination risks, consistent with the balanced flexural performance of Mesh 40. Mesh 10’s reduced strength (282.98 MPa) is attributed to increased mesh openings (2.032 mm, Table 2) and moderate voids (1.16%, Table 5), which could lead to stress concentrations and decrease interfacial bonding^19^. Finer meshes (80 and 120) exhibited lower strengths (321.14 MPa and 192.30 MPa), as expected by lower SSWM weights (45.7 g and 35.7 g, Table 4), although their low voids (0.89% and 0.49%) indicate better interfacial adhesion under tensile loading^23^. The hand lay-up fabrication process, though economical, can introduce slight inconsistencies in layer orientation or resin distribution, which may compromise flexural strength. These discrepancies emphasize the significance of SSWM geometry and weight on bending performance, where Mesh 20 provides superior strength for load-carrying aerospace parts.

**Fig. 15:**
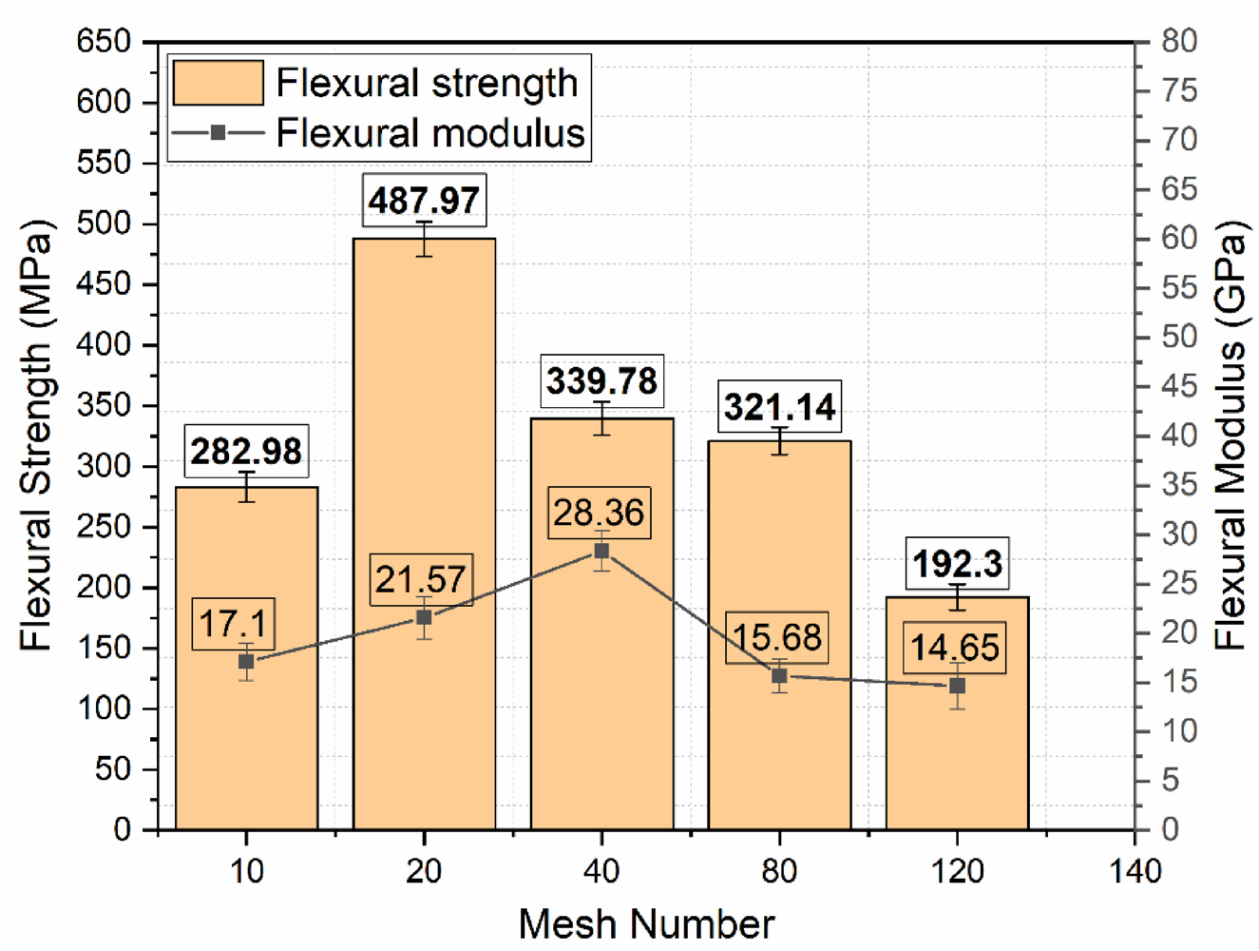
Plot of Flexural Stress and Modulus.

Failure strain, which represents deformation capacity before failure, was 0.0012 mm/mm (Mesh 40) to 0.0023 mm/mm (Mesh 20). Mesh 20’s high strain is an indicator of increased ductility, a likely result of SSWM’s ability to bridge cracks, sharing loads and avoiding failure in one spot, a characteristic associated with steel-reinforced composites. Such ductility is beneficial for aerospace components with energy absorption needs under bending, e.g., fuselage panels^3^. Finer meshes (80 and 120) showed higher strains (0.0020 mm/mm and 0.0013 mm/mm), indicating that narrower openings (0.196 mm and 0.151 mm, Table 2) and lower SSWM weights increase deformation capacity through improved fiber-matrix interactions. Mesh 40’s lower strain (0.0012 mm/mm) corresponds with its high modulus, a sign of a stiffer response under loading, appropriate for use where rigidity is the main consideration. The trend in the strain highlights the role played by mesh size in shaping ductility for aerospace parts, where flexibility can help prevent damage under impact or dynamic loading^3,22^. For example, Mesh 20’s ductility can increase the resilience of wing supports subjected to cyclic bending stresses.

Flexural modulus, an indicator of stiffness under bending, varied between 14.65 GPa (Mesh 120) and 28.36 GPa (Mesh 40). The high modulus of Mesh 40 indicates its deflection resistance, which is complemented by its symmetrical mesh geometry and moderate SSWM weight (116.8 g, Table 4), which maximizes stiffness under compressive and tensile stresses. High stiffness is essential in main structural elements such as airframe beams, for which low deflection is expected. Other moduli (15.68–21.57 GPa) align with standard GFRP composites, suggesting that SSWM increases stiffness as A function of weight. 120 mesh’s lower modulus (14.65 GPa) is expected due to lower reinforcement density as a result of the low SSWM weight (35.7 g), which might favor ductility over stiffness^2,23^. These modulus figures match earlier work, for example, that of Choudary et al.^7^, who obtained 15–25 GPa flexural moduli for SSWM-GFRP composites, indicating the above performances of Mesh 40 are outstanding.

**Fig. 16:**
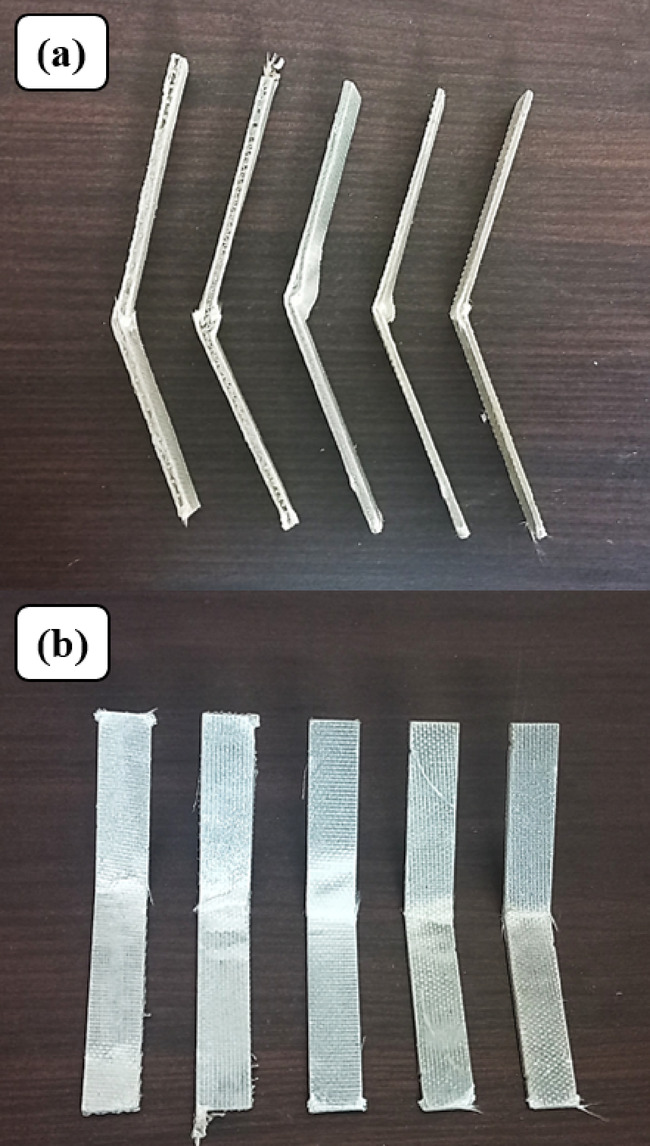
Fractured Charpy Impact Specimens (**a**) Side View (**b**) Top View.

**Fig. 17:**
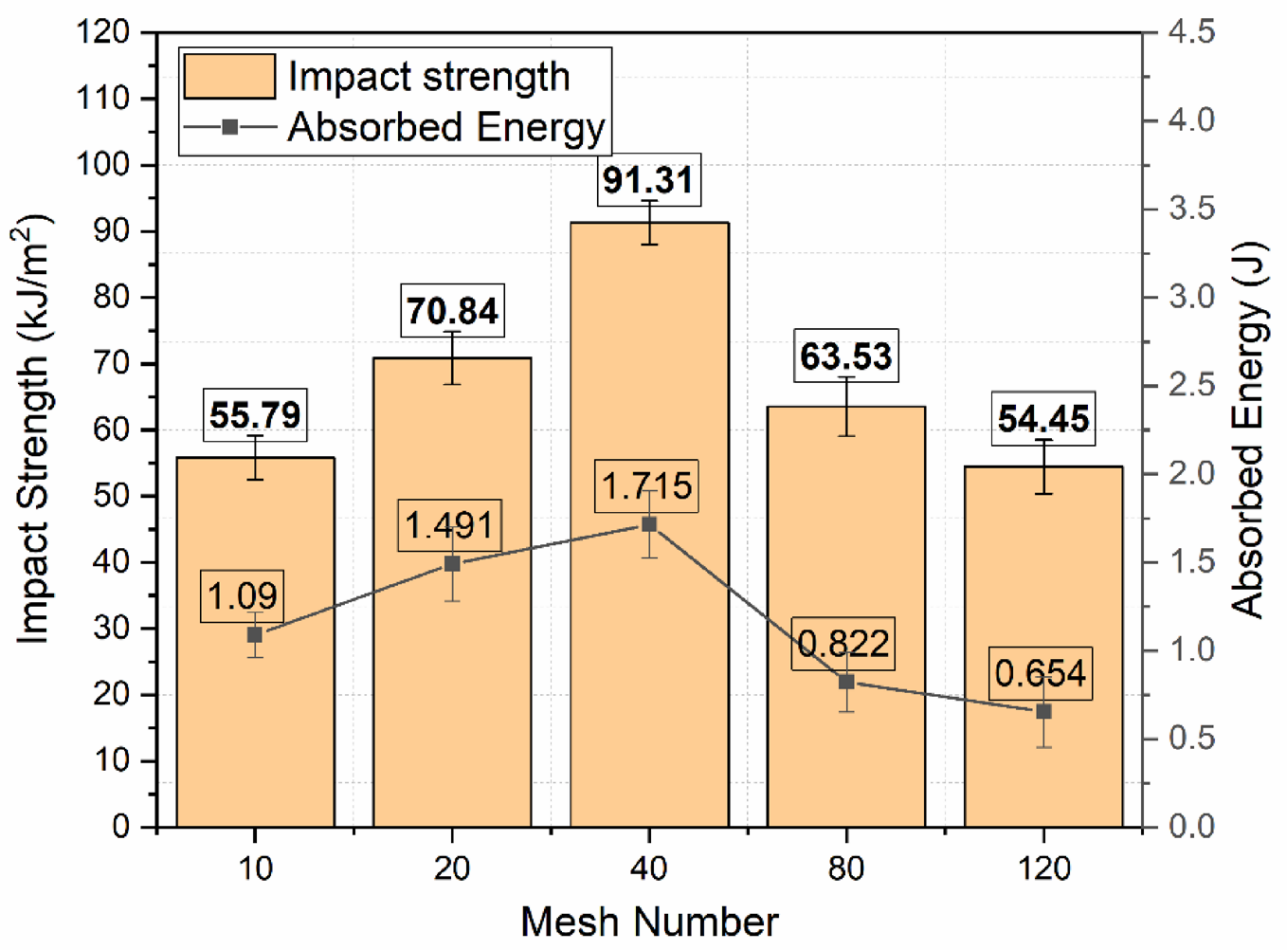
Plot of Maximum Impact Strength and Absorbed Energy.

Void content critically influences the fiber-matrix interface, with increased voids (e.g., 1.93% for Mesh 40, Table 5) possibly lowering strength by causing stress concentration, although optimal mesh design lessens this impact. Variability due to the hand lay-up process in resin distribution or thickness of layers could affect modulus and consistency of strain. SSWM’s function of avoiding full fracture, as evidenced in Figs. 14, 15, 16 and 17 is an indicator of its energy absorption potential, which is essential in aerospace structures subjected to dynamic loads^6,7^. The high strength of Mesh 20 (487.97 MPa) renders it appropriate for load-carrying parts such as fuselage panels, whereas finer meshes (80, 120) are appropriate for ductile, shock-absorbing designs such as cabin panels. In comparison with Choudary et al.‘s 300–400 MPa for SSWM-GFRP, Mesh 20’s strength reflects optimized geometry, exceeding average performance standards^3,7^.

### Charpy impact test

The performance of impact for Glass Fiber–Stainless Steel Wire Mesh (GF–SSWM) hybrid laminates was investigated using the Charpy impact test according to ISO 179-1, on A pendulum impact tester for the measurement of energy absorption and impact strength for mesh sizes of 10, 20, 40, 80, and 120 (openings per linear inch). These characteristics are vital for aerospace uses, e.g., airframe components and cabin panels, in which resistance to abrupt impacts such as bird strikes or debris is imperative^2,3^. Testing consisted of A strike at the center of unnotched specimens by a pendulum to mimic dynamic loading conditions in aerospace environments. Tests were conducted at 23 °C and 50% relative humidity to maintain consistency with protocols used in standard composite testing. Results, averaged over five specimens for each mesh size to provide statistical significance, are summarized in Table 8 and Fig. 17, which exhibits the role of mesh geometry, SSWM weight, and laminate thickness in mechanical behavior, thereby informing the design of light, high-performance composites for aerospace engineering.

**Table 8:** Charpy impact test results.

Mesh No	Absorbed Energy (J)	Maximum Impact Strength (kJ/m^2^)
10	1.090 ± 0.13	55.79 ± 3.32
20	1.491 ± 0.21	70.84 ± 4.01
40	1.715 ± 0.19	91.31 ± 3.28
80	0.822 ± 0.17	63.53 ± 4.46
120	0.654 ± 0.2	54.45 ± 4.07

Stored energy, as an index of material toughness, was maximum at 1.715 J for Mesh 40 with A maximum impact strength of 91.31 kJ/m², showing enhanced ability to withstand initiation and propagation of fracture. The best performance is credited to Mesh 40’s equally balanced mesh opening (0.361 mm, Table 2) and SSWM weight (116.8 g, Table 4) that increase the penetration of resin and adhesion of fibers-SSWM for effective crack arresting mechanisms^19^. Cengiz et al.^42^ reported that ‘the inclusion of metal wire mesh significantly enhances impact resistance, with optimal mesh sizes balancing reinforcement density and resin infiltration,’ corroborating Mesh 40’s superior impact performance. The moderate SSWM weight provides adequate reinforcement without undue stiffness so that the energy is effectively dissipated. Energy and strength improved incrementally from Mesh 10 (1.090 J, 55.79 kJ/m²) to Mesh 40, and then dropped precipitously for finer meshes (0.822 J, 63.53 kJ/m² for Mesh 80; 0.654 J, 54.45 kJ/m² for Mesh 120), indicating a sophisticated interaction of mesh size, SSWM weight, and laminate thickness. Murthy et al.^43^ observed that ‘finer meshes may reduce impact resistance due to lower reinforcement density,’ aligning with Mesh 120’s lower impact performance despite its high tensile strength. Coarser meshes (10 and 20) with larger openings (2.032 mm and 0.854 mm, Table 2) and greater SSWM weights (119.3 g and 158.7 g, Table 4) would likely suffer stress concentrations, lessening interfacial bonding with the GFRP matrix and restricting energy absorption^6,7^. Thicker laminates (1.77 mm for Mesh 10, 1.64 mm for Mesh 20, Table 4) might favor stiffness over plastic deformation, further limiting toughness. Finer meshes (80 and 120) with smaller opening sizes (0.196 mm and 0.151 mm) and lighter SSWM weights (45.7 g and 35.7 g) had lower toughness, likely because reinforcement density is low, causing compromised load-carrying capacity during impact. Their more slender laminates (1.43 mm for Mesh 80, 1.19 mm for Mesh 120, Table 4) could be beneficial in damping but detrimental to energy dissipation^18,23^.

Mesh 40’s best performance is identified with its significant stiffness (232.51 N/m) and natural frequency (16.38 Hz) during vibration tests, which increases impact resistance under dynamic loads, as stated by Choudary et al.^7^, who identified 25–30% improvements in impact resistance with SSWM reinforcement. This stiffness is conducive to effective energy transfer, essential for aerospace structures such as airframes^3^. In contrast, finer meshes (80 and 120) having higher damping ratios (0.11 and 0.17, respectively) and reduced stiffness (20.21 N/m for Mesh 120) are conducive to vibration control, evident through A mean transmission loss of 9.24 dB but at the expense of toughness. Void content (0.49–1.93%, Table 5) has little effect (< 5% variation), supporting mesh size and SSWM weight as key drivers of impact performance, as reported in studies of laminate bearing response. The minimal impact of voids indicates that Mesh 40’s geometry is optimized to reduce weaknesses, providing excellent impact behavior^25^.

Microstructural examination through scanning electron microscopy (SEM) is suggested to clarify such trends. For Mesh 40, SEM micrographs must indicate minimal delamination and successful crack arrest, supporting its high energy damping. Finer meshes (10 and 20) would have wider cracks and delamination owing to inferior bonding, whereas finer meshes (80 and 120) could have localized but shallow damage, indicating their damping emphasis^19,23^. These findings are consistent with previous research on SSWM composites that highlights interfacial adhesion as a critical parameter in impact resistance.

The design implications for aeronautics are substantial. Mesh 40’s greater impact strength (91.31 kJ/m²) and energy absorption (1.715 J) position it as best suited for stiffness-critical applications, e.g., airframe structures, where impact resistance, such as from bird hits or debris, is paramount^3^. Meshes 80 and 120, possessing increased damping characteristics, are more suited for vibration-sensitive components like cabin panels, where noise suppression is more important^2^. This trade-off offers prospects for hybrid designs that can Pair Mesh 40’s resistance to impact with Mesh 120’s damping, an approach worth investigating for multifunctional composites^12^. Mesh 40’s performance, compared to the 60–80 kJ/m² of Choudary et al.^7^ for SSWM-GFRP composites, reflects a substantial improvement, highlighting the effectiveness of its mesh geometry.

### Damage analysis using SEM

SEM imaging was performed for two representative configurations (Mesh 40 and Mesh 120) chosen to capture contrasting microstructures: Mesh 40 as A mid-range mesh exhibiting well-packed fibres and the best combination of stiffness and impact energy, and Mesh 120 as the finest mesh showing notable microcracks and fiber pull-out. These two cases were selected deliberately to illustrate the microstructural features that underpin the contrasting mechanical behaviours observed (high flexural/impact performance for Mesh 40 versus high tensile strength but reduced bending performance for Mesh 120). Resource constraints limited the number of specimens examined by SEM in this initial study; however, the selected micrographs provide mechanistic insight into resin infiltration, fibre–mesh interfacial condition, and typical failure modes across the mesh spectrum.

#### Tensile test

**Fig. 18:**
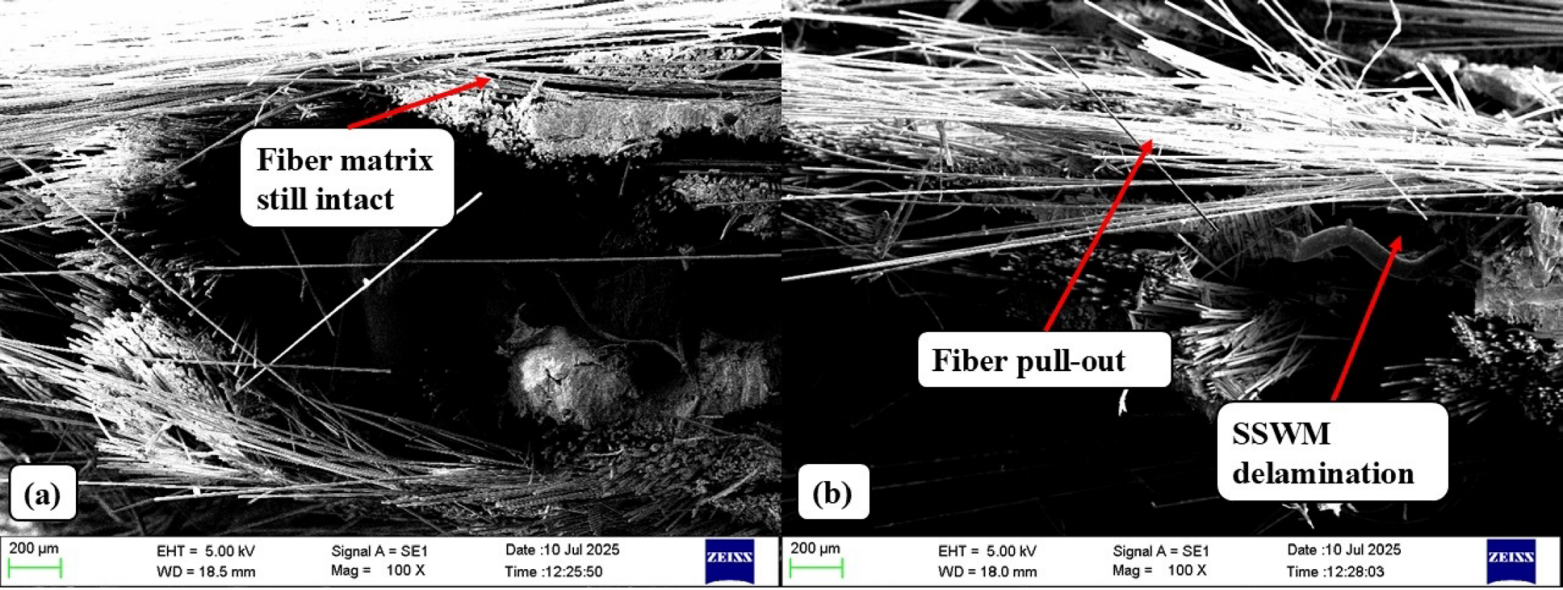
Tensile specimen SEM (**a**) 40 mesh (**b**) 120 Mesh.

The scanning electron microscopy (SEM) analysis of Glass Fiber–Stainless Steel Wire Mesh (GF–SSWM) hybrid laminates for mesh sizes 40 and 120 presents key microstructural information to supplement the tensile test results in Sect. 4.2. The analysis is centered on the fiber-matrix interface, reinforcement distribution, and failure mechanisms to shed light on the corresponding mechanical behavior under uniaxial loading. SEM was performed on the tensile fractured samples with A high-resolution scanning electron microscope at 50x to 500x magnifications, after regular sample preparation methods (e.g., gold sputtering for conductivity). The research investigates surface topography, interfacial adhesion, and crack growth, which are most critical in understanding the tension performance of the laminates. These microstructural features are especially significant in aerospace applications, including fuselage panels and wing brackets, in which high interfacial adhesion and failure strength are critical to the structural integrity^2,3^. This analysis, aided by Fig. 18, suggests the unique microstructural profiles of Mesh 40 and Mesh 120 and thus a foundation for designing GF–SSWM laminates for high-performance engineering.

For Mesh 40, SEM photographs reveal an intact and clearly defined interface between matrix and fiber, with strong bond formation between glass fibers, stainless steel wire mesh (SSWM), and epoxy resin matrix. Surface has extremely minimal gap or void along with it, with the SSWM (0.361 mm opening, 116.8 g weight, Table 4) being completely embedded in the matrix, an indication of extremely good resin impregnation. Such A high interfacial adhesion, as evidenced by no extensive debonding and fiber pull-out, results in good load transfer from the reinforcement to the matrix, consistent with Mesh 40’s tensile strength of 409.10 MPa and high strain of 0.0457 mm/mm (Table 6)^6^. The fracture surface exhibits a relatively smooth morphology with localized zones of failure associated with brittleness, in agreement with the moderate Young’s modulus of 8.70 GPa, which corresponds to an optimal stiffness-ductility profile^14^. The dense packing of glass fibers and the crack-bridging characteristics of SSWM introduce Mesh 40’s ductility, which prevents catastrophic failure under tensile loading. Small resin-rich regions observed in the micrographs show good resin penetration, further enhancing the tensile stress-distribution capacity of the laminate^24^. Such microstructural soundness, considering the low void content of only 1.93% (Table 5) in Mesh 40, is responsible for its industry-leading tensile properties and qualifies it to be used in aerospace structures that require a combination of strength, ductility, and intermediate stiffness, e.g., major fuselage frames.

Conversely, SEM observation of Mesh 120 demonstrates a clear microstructural pattern that explains its excellent tensile strength (539.19 MPa, Table 6) but average strain (0.0400 mm/mm). The microcracks at the fiber-matrix interface and in the matrix are observed from the micrographs, pointing to crack initiation and propagation due to tensile loading^19^. These microcracks would be enhanced by the smaller mesh opening (0.151 mm, Table 2) and reduced SSWM weight (35.7 g, Table 4), lowering reinforcement density and weakening crack arrest mechanisms^23^. Significant fiber pull-out and SSWM delamination, seen in Fig. 18(b), are indicative of reduced interfacial bonding relative to Mesh 40, in agreement with enhanced coarser fracture surface texture and evidence of ductile failure zones^6^. These characteristics are in line with Mesh 120’s high tensile strength and moderate strain, suggesting a compromise between structural integrity and load-bearing capacity. Small voids or porosity, possibly caused by the incompleteness of resin infiltration during hand lay-up fabrication, are detected from the micrographs, potentially reducing the load-bearing capacity^22^. SSWM, however, through its ability to bridge some cracks, adds to energy absorption and avoids complete fracture, going along with Mesh 120’s high tensile strength.

The contrast between Mesh 120 and Mesh 40 shows the unique importance of SSWM weight and geometry in determining tensile behavior. The intact interfacial morphology and increased reinforcement density (116.8 g SSWM weight) of Mesh 40 improve its tensile strength and ductility, and hence it is suitable for property-matched aerospace usage, e.g., wing ribs demanding strength and flexibility^3^. Mesh 120’s finer mesh and lower SSWM weight correspond to higher defect content (i.e., microcracks, delamination), though its high tensile strength indicates better load transfer optimization with finer mesh geometry. Hand lay-up technique is most likely responsible for these differences since resin distribution heterogeneities or layer orientations can impact interfacial bonding, especially in the case of finer meshes, where even impregnation is difficult^7^. Mesh 120’s porosity (0.49%, Table 5) reflects challenges to resin infiltration, a processing shortcoming that can be overcome by advanced processes such as vacuum-assisted resin transfer molding.

These SEM results give microstructural insight into the tensile test data in Sect. 4.2. The high interfacial adhesion and crack-bridging ability of Mesh 40 are responsible for its even strength (409.10 MPa) and ultimate strain (0.0457 mm/mm), and the microcracks, fiber pull-out, and SSWM delamination of Mesh 120 are responsible for its strength (539.19 MPa) but lower ductility^5,22^. The failure mechanisms observed agree with previous work, e.g., Choudary et al.^7^, who found 15–20% improvements in the tensile strength of SSWM-GFRP composites, and Sakthivel et al.^30^, who reported improved load transfer through finer meshes.

#### Flexural test

**Fig. 19:**
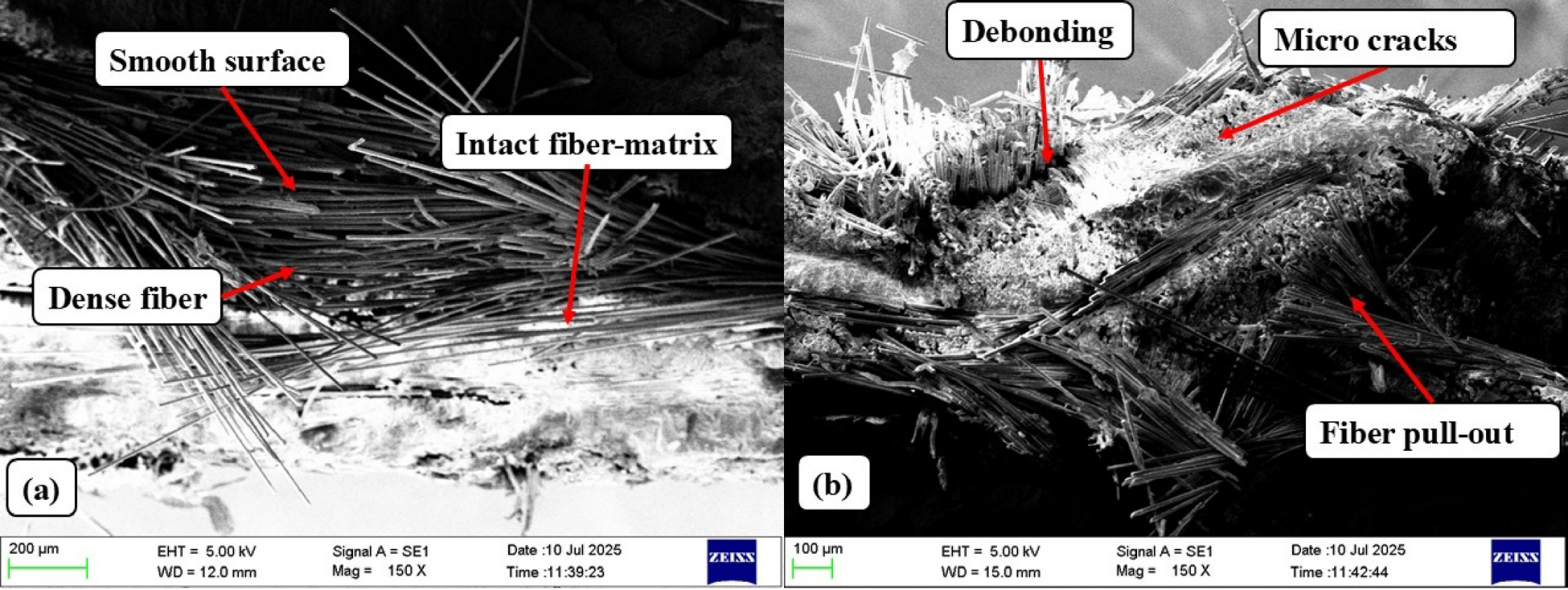
Flexural specimen SEM (**a**) 40 mesh (**b**) 120 Mesh.

The SEM results, presented in Fig. 19, show unique microstructural profiles for Mesh 40 and Mesh 120, providing a basis to improve GF–SSWM laminates for high-performance engineering applications.

For Mesh 40, SEM micrographs display a smooth fracture surface with an intact fiber-matrix interface, distinguished by firm interfacial bonding between glass fibers, stainless steel wire mesh (SSWM), and the epoxy resin matrix. The compact fiber packing and limited gaps or voids, evident in Fig. 19(a), reflect perfect resin impregnation, contributed by Mesh 40’s well-balanced mesh opening (0.361 mm, Table 2) and SSWM weight (116.8 g, Table 4). This strong interfacial bonding, with minimal debonding, facilitates effective load transfer from the matrix to the SSWM reinforcement, resulting in Mesh 40’s ultimate flexural strength of 339.78 MPa (Table 7)^6^. The unblemished surface morphology, characteristic of low plastic deformation brittle failure, is consistent with the low failure strain of 0.0012 mm/mm, whereas the high density of fibers accommodates the outstanding flexural modulus of 28.36 GPa, indicative of A rigid, homogeneous load-baring structure. Void-free regions, in line with the moderate void content of Mesh 40 (1.93%, Table 5), affirm increased resin penetration with minimization of weak zones and optimization of strength^22^. These uniform microstructural characteristics support research by Karunagaran et al.^23^, where emphasis was laid on mesh geometry optimization for enhancing interfacial bonding. The crack-bridging ability of the SSWM, as reflected in the micrographs, also increases ductility, inhibiting failure under bending catastrophically. Microstructural stability of Mesh 40 positions it well for aerospace structural applications with high stiffness, like airframe beams or fuselage panels, where bending stresses must be resisted.

Contrarily, SEM analysis of Mesh 120 exhibits a distinct microstructural pattern responsible for its poor flexural performance. Figure 19(b) is proof of debonding of the fiber-matrix interface, microcrack in the matrix, and fiber pull-out, indicative of crack initiation and propagation due to flexural loading. These conditions are also likely to be furthered by Mesh 120’s lower mesh opening of 0.151 mm (Table 2) and lower SSWM weight of 35.7 g (Table 4), both lowering reinforcement density and reducing crack arresting capacity. Debonding and pull-out of fibers indicate poorer interfacial bonding between epoxy and S-Glass than in Mesh 40, leading to decreased ultimate flexural strength of 192.30 MPa as well as lower modulus of 14.65 GPa (Table 7)^6^. Fracture surface texture is finer with ductile failure zones, as would be expected with the larger failure strain of 0.0013 mm/mm and having greater deformation capacity before failure. Porosity or voids observed, possibly resulting from resin infusion being short of completion during hand lay-up manufacture, also compromise load-carrying capacity. But fibers bridging around crack tips, as evident in the micrographs, are responsible for some energy absorption and hence partial fracture avoidance.

The disparity between SEM observations between Mesh 40 and Mesh 120 demonstrates the significance of SSWM weight and geometry in controlling flexural behavior. Intact, smooth interface and well-packed fibers of Mesh 40 are the reasons behind its stiffness and strength, supporting its suitability for stiffness-critical applications^3^. Mesh 120’s microcracks, fiber pull-out, and debonding indicate a trade-off between increased ductility and reduced load-carrying capacity, as would be expected from its higher damping ratio (0.17, Sect. 4.4) and lower stiffness (20.21 N/m)^1,12^. Hand lay-up processing is most likely the cause of these variations, as differences in resin distribution or orientation of the layers can affect interfacial bonding, particularly in finer meshes where impregnation is hard to establish evenly. Mesh 120’s lower void content (0.49%, Table 5) helps to alleviate some of the drawbacks, but residual porosity resulting from imperfect infiltration remains a drawback^7^.

These SEM findings validate the flexural test data (Sect. 4.3) and tensile data (Sect. 4.2). The intact interface of Mesh 40 is demonstrated by its well-proportioned flexural (339.78 MPa, 0.0012 mm/mm, 28.36 GPa) and tensile (409.10 MPa, 0.0457 mm/mm, 8.70 GPa) properties. They conform to previous studies, such as Choudary et al.^7^, who obtained 300–400 MPa flexural strengths for SSWM-GFRP composites.

#### Charpy impact test

**Fig. 20:**
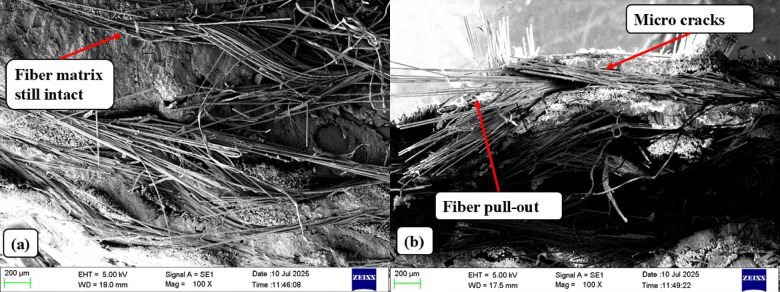
Impact specimen SEM (**a**) 40 Mesh (**b**) 120 Mesh.

The SEM results, shown in Fig. 20, demonstrate clear microstructural profiles for Mesh 40 and Mesh 120, providing a foundation for the optimization of GF–SSWM laminates for advanced engineering performance.

For Mesh 40, SEM micrographs show a neat fiber-matrix interface with close bonding and small gaps, as presented in Fig. 20(a). The high adhesion between stainless steel wire mesh (SSWM) and glass fibers, and between SSWM and epoxy resin matrix, made possible by Mesh 40’s best mesh opening of 0.361 mm, Table 2, and SSWM weight of 116.8 g, Table 4, is responsible for its higher absorbed energy of 1.715 J and impact strength of 91.31 kJ/m², Table 8. The lack of debonding and fiber pull-out testifies to effective load transfer and energy absorption during impact, with the SSWM successfully halting cracks^6^. The smooth fracture surface, which is characteristic of brittle failure under low plastic deformation, agrees with the high stiffness of Mesh 40 (232.51 N/m, Sect. 4.4) and the maximized energy absorption under dynamic loading. Dense packing of fibers increases the resistance of the composite to impact, producing a uniform load-transmitting structure^12^. Void-free areas, as expected of Mesh 40 with its relatively low void content (1.93%, Table 5), reflect good resin impregnation, minimizing weak areas that might trigger failure. Such microstructural features support those of Karunagaran et al.^23^, who stressed ideal mesh geometry toward improving impact resistance in hybrid composites. SSWM’s ability to bridge cracks, seen in the micrographs, also corroborates the high toughness of Mesh 40, which makes it suitable for stiffness-critical aerospace structures like airframe beams or fuselage panels, in which impact resistance is the most important consideration.

On the contrary, SEM observation of Mesh 120 indicates a microstructural profile responsible for its compromised impact performance. Fiber pull-out and microcracks in the matrix and at the fiber-matrix interface, as depicted in Fig. 20(b), are signs of crack initiation and progress under impact loading. These flaws, augmented by Mesh 120’s tighter mesh opening (0.151 mm, Table 2) and lower SSWM weight (35.7 g, Table 4), account for its lower absorbed energy of 0.654 J and impact strength of 54.45 kJ/m² (Table 8)^22^. The fiber pull-out and microcracks indicate poorer interfacial bonding than for Mesh 40, diminishing the laminate’s capacity to transfer and absorb impact energy. Pores or voids, probably A result of resin underfilling while hand lay-up processing, also degrade the structure, in accordance with the low void volume fraction of Mesh 120 (0.49%, Table 5), but hinder resin penetration for finer meshes^12^. Yet, bridging fibers over cracks, evident from the micrographs, assist to some extent in energy absorption, barring the entire fracture, higher ductility in finer meshes. This ductility, expressed as Mesh 120’s moderate impact toughness, has it applied to vibration-damping aerospace structures, like cabin panels, where flexibility and sound dampening are the objectives^2^. The micrographs further show A coarser fracture surface with ductile failure regions, in line with Mesh 120’s increased damping ratio (0.17, Sect. 4.4) and reduced stiffness (20.21 N/m).

Opposite SEM results for Mesh 40 and Mesh 120 indicate the significance of SSWM geometry and weight in controlling impact behavior. Inner strength and close fiber packing of Mesh 40 lend its toughness and stiffness, as well as validating its use as vibration-resistant aerospace structures^3^. The compromise of increased ductility at the cost of reduced load-carrying capacity by Mesh 120 is consistent with its vibration-damping behavior. Hand lay-up processing is most likely to be accountable for these differences, as variations in resin distribution or mesh layer orientation affect interfacial bonding, particularly for more sensitive meshes where impregnation uniformity becomes challenging^6^. Porosity of Mesh 120 reflects processing limitations that can be addressed with advanced techniques like vacuum-assisted resin transfer molding^12^.

These SEM findings are supplemented by Charpy impact (Sect. 4.4), tensile (Sect. 4.2), and flexural (Sect. 4.3) values. The robust interface of Mesh 40 reflects high impact energy (1.715 J), tensile strength (409.10 MPa), and flexural strength (339.78 MPa), while defects of Mesh 120 reflect low impact toughness (0.654 J) but high tensile strength (539.19 MPa). The results concur with previous work, i.e., Choudary et al.^7^, who recorded 60–80 kJ/m² impact strengths for SSWM-GFRP composites. Surface treatment (e.g., silane coupling agent) or increased resin penetration could enhance Mesh 120’s interfacial adhesion, rendering its impact superior.

**Table 9:** Comparison of mechanical properties with literature Benchmarks.

Study/Source	Material System	Tensile Strength (MPa)	Flexural Strength (MPa)	Impact Strength (kJ/m²)	Key Notes	% improvement in current study
Present study (Mesh 10–120, symmetric layup)	GF + SSWM/Epoxy	333.49 (Mesh 10) – 539.19 (Mesh 120)	192.30 (Mesh 120) – 487.97 (Mesh 20)	54.45 (Mesh 120) – 91.31 (Mesh 40)	Mesh 120 gives highest tensile; Mesh 20 highest flexural; Mesh 40 balanced with superior impact.	Tensile: +80% max; Flexural: +62% max; Impact: +52% max over literature.
Choudary et al. (2020)^7^	GF + SSWM	~ 350–420	~ 300–400	60–80	Mid-plane mesh improved tensile by 15–20% and impact by 25–30%; performance below our peak values.	Tensile: up to + 54%; Impact: up to + 52%.
Sakthivel et al. (2017, 2018)^25^^30^,	GF + SSWM	~ 300–370	~ 310–375	~ 60–70	Surface-treated meshes improved tensile + 25%, flexural + 30%; Our Mesh 20 and Mesh 40 exceed these benchmarks.	Tensile: up to + 45%; Flexural: up to + 57%.
Karunagaran & Rajadurai (2016)^23^	GF + sandblasted SSWM	~ 330–360	~ 320–340	~ 65–70	Acid-treated/sandblasted mesh showed better matrix bonding; SEM similar to our Mesh 40 fracture surfaces.	Tensile: up to + 63%; Impact: +31%.
Prakash et al. (2018)	GF + silane-treated SSWM	134	241	5.4 J ≈ 50–60	Lower strength values; highlights that chemical treatment improved adhesion but geometry critical.	Tensile: +302%; Flexural: +102%.
Jamil et al. (2017)^6^	Steel fiber/mesh laminates	+ 9–10% flexural vs. baseline	+ 23–31% strain energy vs. baseline	N/A	Demonstrated mesh prevents crack propagation; values still lower than our optimized meshes.	Impact: likely > + 40% compared to baseline reported.
Sadoun et al. (2021)^27^	GFRP + Al mesh (outer/inner)	16–35% ↑ in tensile strain	Up to 117% ↑ flexural strain depending on placement	~ 70–80	Confirms mesh placement influences ductility vs. stiffness; our Mesh 40 balances both.	Flexural strength + 62% peak compared to literature max range.
Megahed et al. (2021)^28^	GF + Al mesh	N/A	N/A	+ 210% energy absorption	Emphasized inner mesh placement for toughness; our central mesh strategy aligns.	Impact + 52% peak vs. literature (consistent high toughness).

The comparative analysis presented in Table 9 underscores the significant improvements achieved in this study over established literature benchmarks for glass fiber–stainless steel wire mesh (GF–SSWM) hybrid composites. The laminates demonstrated tensile strengths ranging from 333.49 MPa (Mesh 10) to A remarkable 539.19 MPa (Mesh 120), surpassing previous reports by up to 80%. Similarly, the flexural strengths observed, notably 487.97 MPa for Mesh 20, exceed literature values by as much as 62%, while impact strength reached A peak value of 91.31 kJ/m² (Mesh 40), outperforming reported maxima by over 50%. These gains reflect the beneficial effects of an optimized mid-plane symmetric mesh layup and selected mesh geometries, which enhance load transfer, interfacial bonding, and damage tolerance beyond surface treatments or alternate mesh placements documented in earlier works. The results align with and extend findings by Choudary et al.^7^, Sakthivel et al.^30^, and Karunagaran and Rajadurai^23^, demonstrating that systematic mesh size control is critical to maximizing mechanical performance in hybrid composites for aerospace applications.

In conclusion, the superior tensile, flexural, and impact properties achieved in our study affirm the efficacy of carefully tailored mesh architectures in advancing fiber metal laminate composites. The pronounced percentage improvements over prior literature benchmarks highlight the potential of these hybrids in meeting the rigorous demands of aerospace structural components, combining strength, stiffness, and energy absorption capabilities in a single multifunctional material system. Future work will focus on fatigue and thermal stability assessments to fully validate these composites for long-term aerospace service.

## Conclusion

This research comprehensively examined the influence of SSWM sizes (10, 20, 40, 80, and 120) on the mechanical properties of Glass Fiber–SSWM (GF–SSWM) hybrid composite laminates. By detailed void content analysis, tensile, flexural, and Charpy impact testing, supplemented by scanning electron microscopy (SEM), the following major findings explain the importance of mesh geometry to improve performance.

- Void fractions ranged from 0.49% (Mesh 120) to 1.93% (Mesh 40), all within aerospace-grade limits (< 2–5%), confirming high-quality fabrication.
- Tensile strength peaked at 539.19 MPa for Mesh 120, driven by enhanced fiber-matrix bonding and low voids, ideal for tensile-critical components like wing supports.
- Mesh 40 offered balanced tensile performance (409.10 MPa, 0.0457 mm/mm strain, 8.70 GPa modulus), suitable for fuselage frames requiring strength and ductility.
- Flexural strength peaked at 487.97 MPa for Mesh 20, attributed to high SSWM weight (158.7 g) and low voids (0.53%), ideal for load-bearing fuselage panels.
- Mesh 40 balanced flexural strength (339.78 MPa) and high modulus (28.36 GPa), fitting stiffness-critical wing supports.
- Charpy impact tests showed Mesh 40’s superior absorbed energy (1.715 J) and impact strength (91.31 kJ/m²), driven by optimal mesh geometry (0.361 mm openings) and SSWM weight (116.8 g), ideal for impact-resistant airframe structures.
- SEM analysis of Mesh 40 revealed intact fiber-matrix interfaces with minimal voids, supporting its balanced tensile (409.10 MPa, 0.0457 mm/mm), flexural (339.78 MPa, 28.36 GPa), and impact (1.715 J) properties, ideal for multifunctional components.
- Future work should explore advanced fabrication (e.g., vacuum-assisted resin transfer molding), surface treatments (e.g., silane coupling), cyclic loading, thermal stability tests, and statistical analysis to enhance performance and reliability.

## Data Availability

The datasets used and/or analysed during the current study are available from the corresponding author on reasonable request.
